# Cerebrospinal fluid quinolinic acid is strongly associated with delirium and mortality in hip-fracture patients

**DOI:** 10.1172/JCI163472

**Published:** 2023-01-17

**Authors:** Leiv Otto Watne, Christian Thomas Pollmann, Bjørn Erik Neerland, Else Quist-Paulsen, Nathalie Bodd Halaas, Ane-Victoria Idland, Bjørnar Hassel, Kristi Henjum, Anne-Brita Knapskog, Frede Frihagen, Johan Raeder, Aasmund Godø, Per Magne Ueland, Adrian McCann, Wender Figved, Geir Selbæk, Henrik Zetterberg, Evandro F. Fang, Marius Myrstad, Lasse M. Giil

**Affiliations:** 1 Oslo Delirium Research Group, Oslo University Hospital, Oslo, Norway.; 2 Institute of Clinical Medicine, Campus Ahus, University of Oslo, Oslo, Norway.; 3 Department of Geriatric Medicine and; 4 Department of Orthopedic Surgery, Akershus University Hospital, Lørenskog, Norway.; 5 Department of Microbiology, Oslo University Hospital, Oslo, Norway.; 6 Department of Anesthesiology, Akershus University Hospital, Lørenskog, Norway.; 7 Department of Neurohabilitation, Oslo University Hospital, Oslo, Norway.; 8 Institute of Clinical Medicine, University of Oslo, Oslo, Norway.; 9 Department of Geriatric Medicine, Oslo University Hospital, Oslo, Norway.; 10 Department of Orthopaedic Surgery, Østfold Hospital Trust, Grålum, Norway.; 11 Department of Anesthesiology, Oslo University Hospital, Oslo, Norway.; 12 Department of Anesthesiology, Diakonhjemmet Hospital, Oslo, Norway.; 13 Bevital AS, Bergen, Norway.; 14 Orthopaedic Department, Bærum Hospital, Vestre Viken Hospital Trust, Drammen, Norway.; 15 Norwegian National Centre for Ageing and Health, Vestfold Hospital Trust, Tønsberg, Norway.; 16 Department of Psychiatry and Neurochemistry, Institute of Neuroscience and Physiology, Sahlgrenska Academy at the University of Gothenburg, Mölndal, Sweden.; 17 Clinical Neurochemistry Laboratory, Sahlgrenska University Hospital, Mölndal, Sweden.; 18 Department of Neurodegenerative Disease, UCL Institute of Neurology, Queen Square, London, United Kingdom.; 19 UK Dementia Research Institute at UCL, London, United Kingdom.; 20 Hong Kong Center for Neurodegenerative Diseases, Clear Water Bay, Hong Kong, China.; 21 Department of Clinical Molecular Biology, University of Oslo, and Akershus University Hospital, Lørenskog, Norway.; 22 The Norwegian Centre on Healthy Ageing (NO-Age), Oslo, Norway.; 23 Department of Internal Medicine, Bærum Hospital, Vestre Viken Hospital Trust, Drammen, Norway.; 24 Neuro-SysMed, Department of Internal Medicine, Haraldsplass Deaconess Hospital, Bergen, Norway.; 25 Department of Clinical Science, University of Bergen, Bergen, Norway.

**Keywords:** Inflammation, Metabolism, Dementia, Neurological disorders, Psychiatric diseases

## Abstract

**BACKGROUND:**

The kynurenine pathway (KP) has been identified as a potential mediator linking acute illness to cognitive dysfunction by generating neuroactive metabolites in response to inflammation. Delirium (acute confusion) is a common complication of acute illness and is associated with increased risk of dementia and mortality. However, the molecular mechanisms underlying delirium, particularly in relation to the KP, remain elusive.

**METHODS:**

We undertook a multicenter observational study with 586 hospitalized patients (248 with delirium) and investigated associations between delirium and KP metabolites measured in cerebrospinal fluid (CSF) and serum by targeted metabolomics. We also explored associations between KP metabolites and markers of neuronal damage and 1-year mortality.

**RESULTS:**

In delirium, we found concentrations of the neurotoxic metabolite quinolinic acid in CSF (CSF-QA) (OR 2.26 [1.78, 2.87], *P* < 0.001) to be increased and also found increases in several other KP metabolites in serum and CSF. In addition, CSF-QA was associated with the neuronal damage marker neurofilament light chain (NfL) (β 0.43, *P* < 0.001) and was a strong predictor of 1-year mortality (HR 4.35 [2.93, 6.45] for CSF-QA ≥ 100 nmol/L, *P* < 0.001). The associations between CSF-QA and delirium, neuronal damage, and mortality remained highly significant following adjustment for confounders and multiple comparisons.

**CONCLUSION:**

Our data identified how systemic inflammation, neurotoxicity, and delirium are strongly linked via the KP and should inform future delirium prevention and treatment clinical trials that target enzymes of the KP.

**FUNDING:**

Norwegian Health Association and South-Eastern Norway Regional Health Authorities.

## Graphical abstract

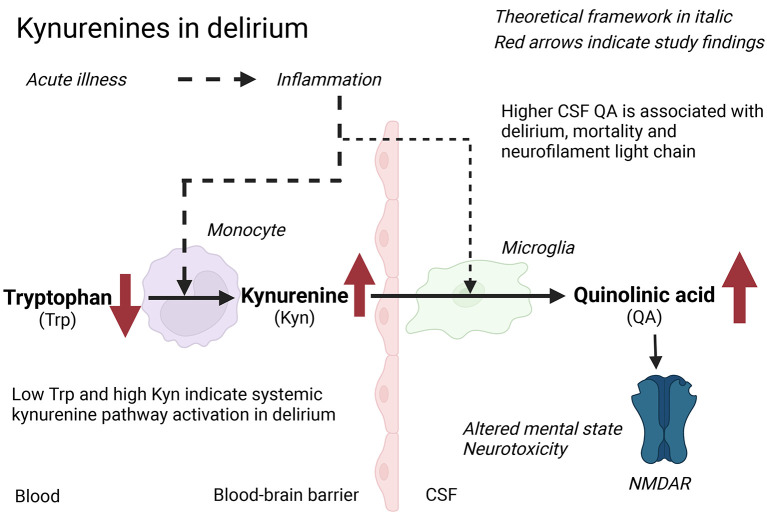

## Introduction

The kynurenine pathway (KP) has been identified as a potential mediator linking acute illness to cognitive dysfunction by generating neuroactive metabolites in response to inflammation (1, 2). An imbalance of KP intermediates, exerting either neuroprotective or neurotoxic effects, has been linked to inflammation, neuronal damage, and ultimately a variety of neurodegenerative and psychiatric diseases (3, 4). Under physiological conditions, the first step in the KP is regulated by the rate-limiting enzyme tryptophan 2,3-dioxygenase (TDO), which catalyzes the conversion of tryptophan (Trp) to kynurenine (Kyn), subsequently leading to the production of several intermediates and end products, including the neuroprotective kynurenic acid (KA) and picolinic acid (Pic) as well as the neurotoxic 3-hydroxykynurenine (HK), 3-hydroxyanthranilic acid (HAA), and quinolinic acid (QA) (Supplemental Figure 1; supplemental material available online with this article; https://doi.org/10.1172/JCI163472DS1). However, during systemic inflammation, proinflammatory cytokines induce indoleamine 2,3 dioxygenase (IDO) expressed in monocytes and dendritic cells, which also catalyzes the conversion of Trp to Kyn (5). Kyn is capable of crossing the blood-brain barrier (BBB) and acts as the precursor for approximately 60% of KP metabolites in the brain. Thus, systemic activation of IDO may be capable of increasing brain concentrations of metabolites such as the *N*-methyl-d-aspartate receptor (NMDAR) antagonist KA and the NMDAR agonist QA (6, 7). The NMDAR is expressed ubiquitously in the nervous system, but mostly in the cortex and hippocampus (8). This could be of relevance to the pathogenesis of delirium, as the NMDAR is implicated in cognitive function, psychosis, and excitotoxicity-induced neuronal cell death (2).

Delirium, a frequent and severe complication of acute illness, is characterized by sudden impairments in awareness and cognition (9). Despite its high prevalence in acutely hospitalized patients and a significant risk for mortality and future dementia (9, 10), the pathophysiology of delirium remains largely unknown. The recent COVID-19 pandemic further highlighted delirium, as it is a common consequence of severe cases of COVID-19 (11). Proposed mechanisms of delirium include transient disruptions in neuroinflammatory and neurotransmitter pathways (9, 12). We propose that systemic inflammation, triggered by, e.g., tissue damage or infection activates IDO, resulting in disrupted balance of the KP metabolites and, ultimately, delirium. Indeed, experimental studies have implicated the KP as a mediator of inflammation-induced cognitive dysfunction. In rats subjected to polymicrobial sepsis, IDO inhibitors prevented cognitive impairment (13). Similarly, IDO-deficient mice were protected from cognitive dysfunction following endotoxin injection (6). Clinical studies have identified an increased Kyn/Trp ratio (KTR) in blood, a potential marker of IDO activity that usually increases during inflammation, to be associated with delirium (14–16). However, to the best of our knowledge, no studies have been conducted on KP metabolites in cerebrospinal fluid (CSF), and the potentially neuroactive metabolites have not been measured, thus leaving unresolved the key issue of the potential for engagement of neuroactive KP metabolites with their targets in delirium.

We investigated the association between delirium and KP metabolite concentrations in CSF and serum in acutely hospitalized patients. We chose patients diagnosed with hip fracture, as this group commonly develops delirium, and CSF sampling is feasible in conjunction with preoperative spinal anesthesia (17, 18). In addition, we included a group of patients with delirium triggered by another medical condition to compare KP metabolite concentrations in relation to different etiologies of delirium. Cognitively unimpaired adults without delirium or dementia served as a reference group. To explore the pathophysiological relevance of the KP to delirium and outcomes for hip-fracture patients, we also investigated the association between KP metabolites and the neuronal damage marker neurofilament light chain (NfL) (19) and 1-year mortality. We hypothesized that the KP in CSF and blood was upregulated in delirium and that an accumulation of neuroactive metabolites was associated with delirium.

## Results

### Demographics and clinical characteristics.

**Figure 1:**
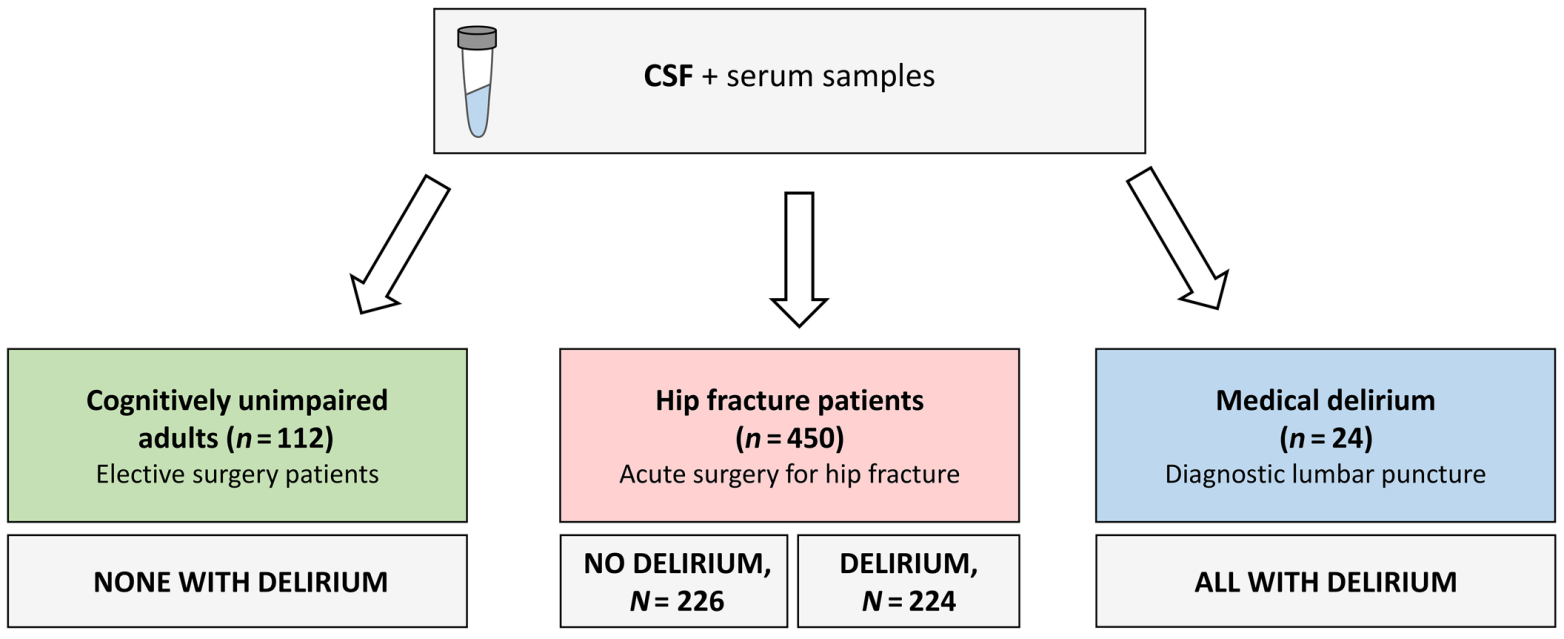
Overview of study design and included patients. We undertook a multicenter observational study with 586 hospitalized patients and investigated associations between delirium and KP metabolites measured in CSF and serum by targeted metabolomics. We also explored associations between KP metabolites and markers of neuronal damage and 1-year mortality. Patients from 2009 to 2019 were included, and CSF samples were available from 450 hip-fracture patients (224 with and 226 without delirium), 24 participants from an independent cohort of patients with medical delirium, and 112 cognitively unimpaired adults scheduled for elective surgery under spinal anesthesia. Serum samples collected at the same time as CSF were available from 338 patients. Delirium was assessed daily according to DSM-5 criteria and based on a standardized procedure. See Supplemental Appendix.

We undertook a multicenter observational study enrolling 586 participants, with CSF and serum samples collected for metabolic studies. These participants were from 3 study cohorts (*P* values in text); key demographics and clinical characteristics are summarized in Figure 1 and Table 1. The hip-fracture patients were older and more often women (mean age: yr [SD] 81 [11], 32% male, 68% female) compared with the medical delirium patients (69 [12], 58% male, 42% female) and the cohort of cognitively unimpaired adults (73 [7], 55% male, 45% female) (both *P* < 0.001, for both *t* test and Pearson’s *x*^2^). Delirium occurred in 224 (49.8%) hip-fracture patients during the hospital stay. Of these, 113 had delirium before surgery (prevalent) and 108 after surgery (incident), while this information was missing for 3 patients. An additional 44 patients had subsyndromal delirium (SSD). Hip-fracture patients with delirium more often had prefracture cognitive impairment (Informant Questionnaire on Cognitive Decline in the Elderly [IQCODE] ≥ 3.44, 72% versus 19%; ref. 20), and more had severe systemic disease (American Society of Anesthesiologists [ASA] physical status score III–IV, 68% versus 39%; see Table 1 legend for explanation of ASA scores (21) compared with hip-fracture patients without delirium (both *P* < 0.001, Pearson’s *x*^2^).

### KP metabolites and associations with delirium.

**Figure 2:**
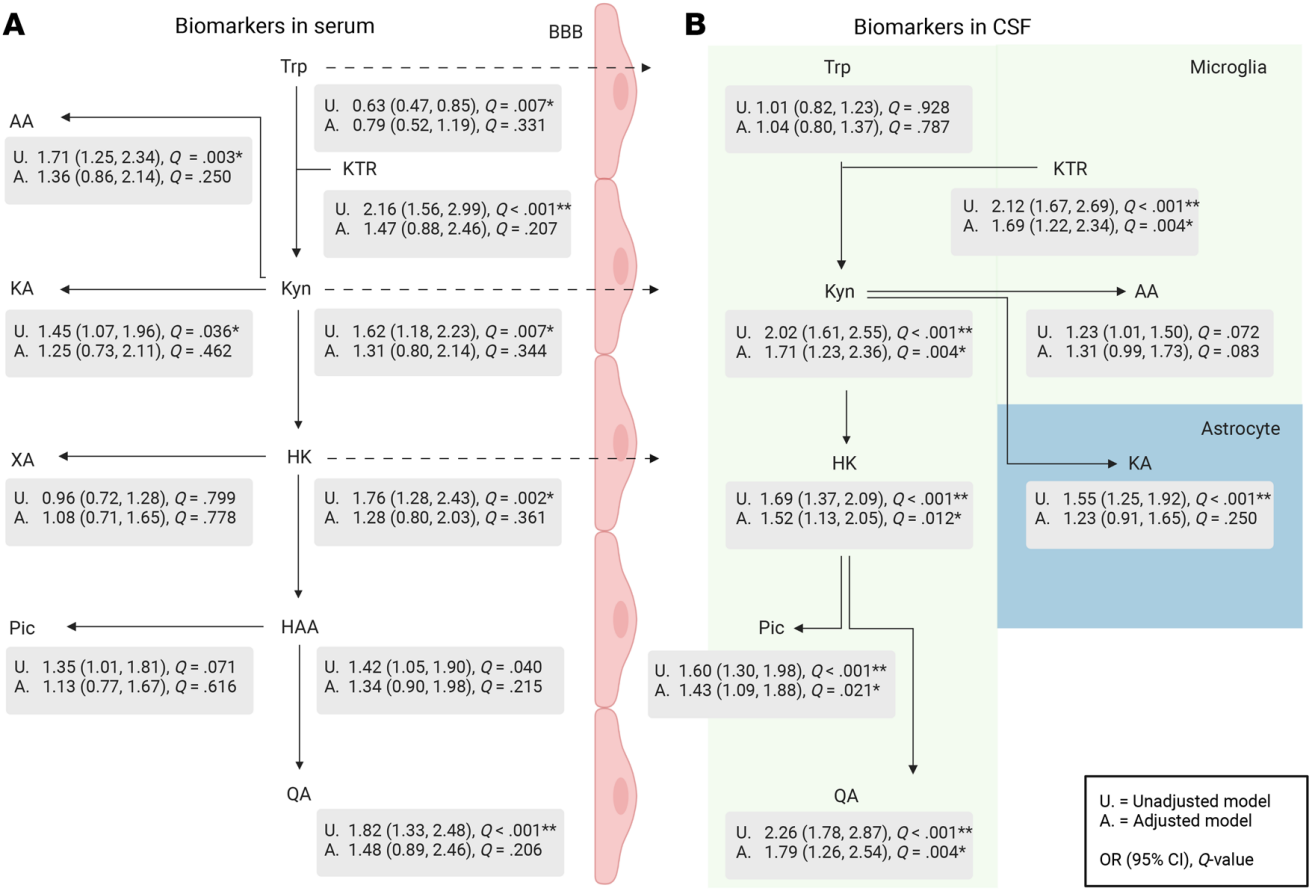
Trp, KP metabolites, and delirium in the hip-fracture cohort. Unadjusted and adjusted logistic regression with delirium as the outcome in the hip-fracture cohort (224 with delirium and 182 without delirium; 44 with SSD excluded from analysis). Serum metabolites (**A**, *n* = 213) and CSF metabolites (**B**, *n* = 406) were both associated with delirium, although CSF metabolites (**B**) were in general more strongly associated with delirium and remained so in adjusted analyses. The strongest association was with CSF QA. Covariates were age, sex, renal function (GFR), ASA score (I–II versus III–IV), and IQCODE (≥ 3.44 versus < 3.44). Serum Trp was untransformed; CSF Trp, Kyn, HK, Pic, and QA were transformed using the inverse square root transformation. All other metabolites were log transformed. Following transformation, the metabolites were standardized to a mean of 0 and an SD of 1. Inverse square root transformations generally provide somewhat lower ORs than log transformations. *Q < 0.05; **Q < 0.001 (*Q* values are *P* values adjusted for multiple comparisons so that *Q* < 0.05 indicates significance after adjustment for multiple comparisons).

Figure 2 lists the ORs for delirium in hip-fracture patients according to concentrations of serum and CSF KP metabolites (please also see Supplemental Table 1 for effect sizes). In unadjusted analyses, several KP metabolites in both serum and CSF were significantly associated with delirium. Importantly, only KP metabolites in the CSF remained significant after adjustment for age, sex, glomerular filtration rate (GFR), IQCODE, and ASA score. Although the KP metabolites were associated with sex, GFR, IQCODE, and ASA score, the effect sizes were most attenuated by age, which was the most important confounder (Supplemental Table 2).

**Figure 3:**
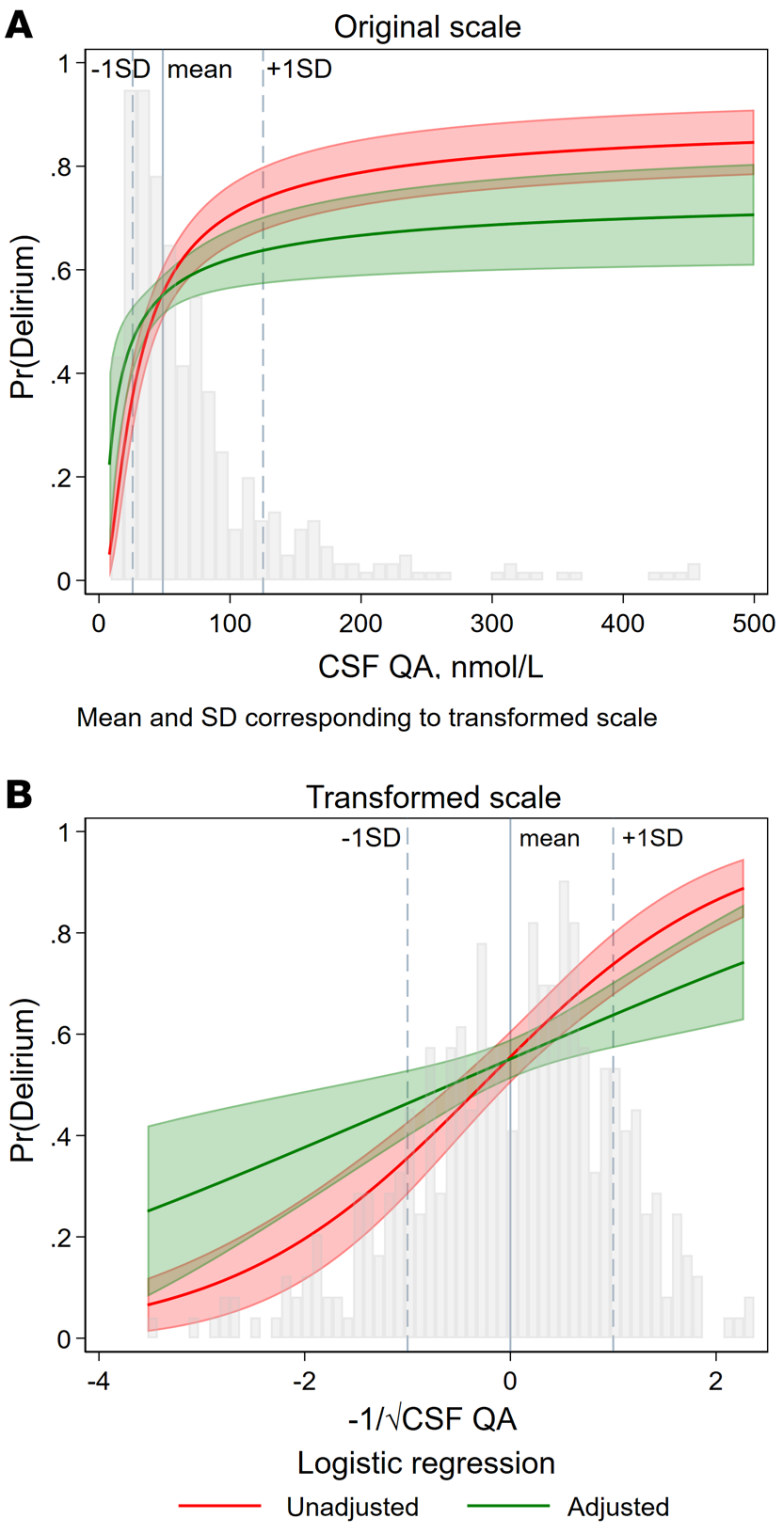
QA concentrations in CSF and delirium. (**A**) The form of the effect size of the QA-delirium association using logistic regression is depicted on the nmol/L scale (*n* = 406, 224 with delirium and 182 without delirium, 44 with SSD excluded). The marginal predictions from logistic regression using transformed CSF-QA (**B**) have been back-transformed to the original scale of CSF-QA in nmol/L in unadjusted (red) and adjusted (green) analyses, with age, sex, GFR, ASA score (I–II versus III–IV), and IQCODE (≥3.44 versus <3.44) as covariates. In **A**, CSF-QA values greater than 500 nmol/L (*n* = 4) have been left out for illustrative purposes, but were included in the statistical analyses. In the background, one can see the highly skewed distribution that has been transformed to approximate normality (**B**). On the transformed scale (**B**), a 1 SD increase in CSF-QA gives an OR of 2.26 (unadjusted) and 1.79 (adjusted) for delirium (Figure 1). However, this is highly nonlinear on the nmol/L scale (**A**), where the per unit effect of CSF-QA on the probability (Pr) of delirium decreases incrementally as CSF-QA concentrations increase. This was confirmed using per 50 nmol/L and per 100 nmol/L quantitative cutoffs as predictors of delirium (data not shown).

Of the KP metabolites, QA in CSF (CSF-QA) was most strongly associated with delirium (Supplemental Table 3 lists the adjusted logistic regression model including QA and the covariates). On the nmol/L scale, the effect declined with higher CSF-QA values (Figure 3). The median CSF-QA concentrations (Table 2) in cognitively unimpaired adults and hip-fracture patients without delirium were similar (39 and 41 nmol/L, respectively), but substantially higher in both patient groups with delirium (hip fracture, 69 nmol/L; medical delirium, 84 nmol/L). Despite a strong CSF/serum correlation, CSF-QA was a stronger predictor of delirium than serum QA (Figure 2 and Supplemental Table 4).

### QA, other NMDAR agonists, and potential neurotoxicity.

**Figure 4:**
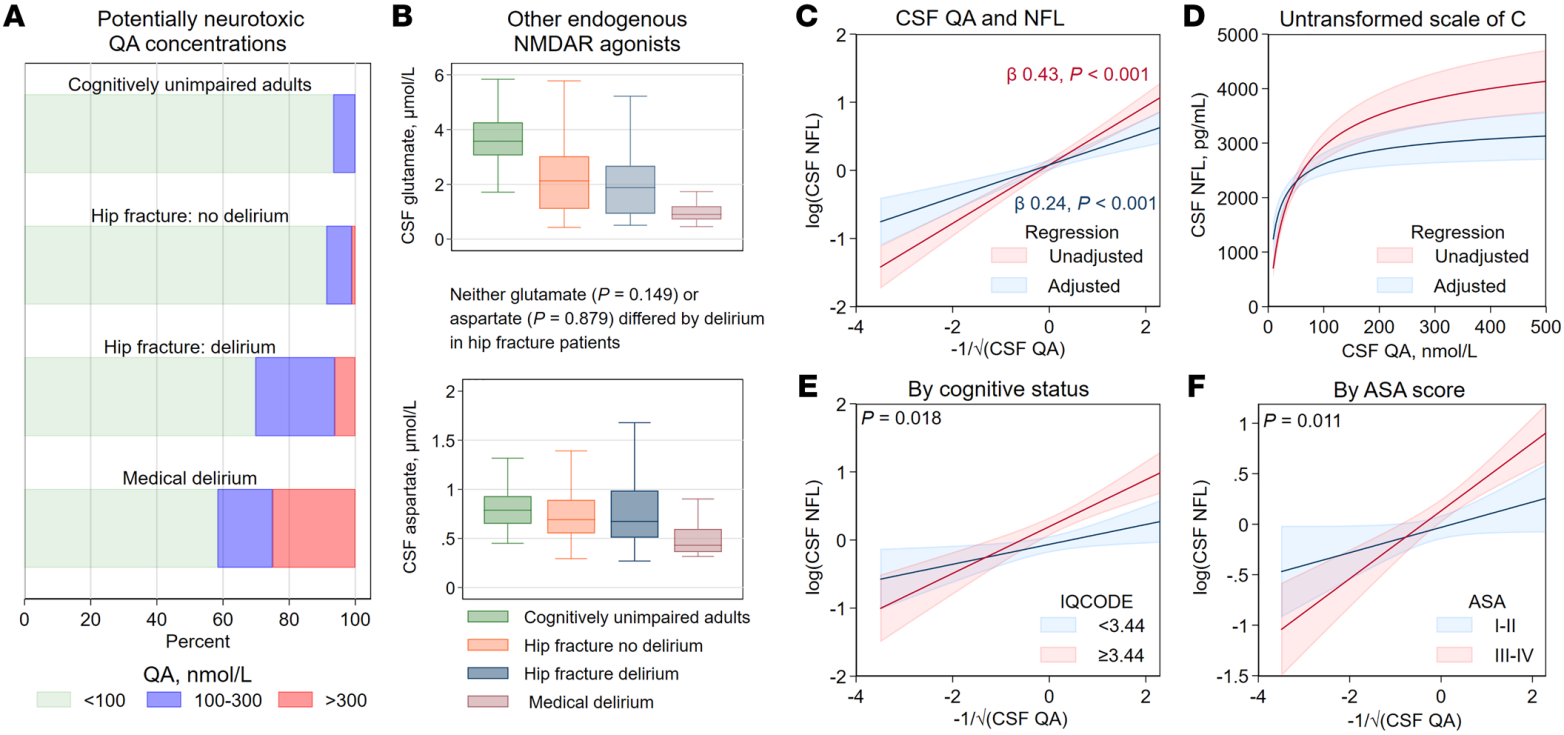
QA, NMDAR agonists, and potential neurotoxicity. Potentially neurotoxic concentrations of CSF-QA, although observed in a minority of delirium patients, were much more frequent in delirium (**A**). Glutamate and aspartate, like QA, stimulate the NMDAR. Although there was no significant difference in CSF glutamate and aspartate concentrations in hip-fracture patients by delirium presence, the overall tendency was for the highest concentrations to occur in cognitively unimpaired adults and the lowest concentrations in medical delirium (**B**). QA was significantly associated with the neuronal injury marker NfL in hip-fracture patients using standardized linear regression (**C** and **D**), also adjusted for age, sex, renal function, and cognitive impairment (IQCODE ≥ 3.44), ASA score (I–II versus III–IV), and delirium status (standardized linear regression with an interaction). Data shown in **A** and **B** included 406 hip-fracture patients (excluding SSD), 112 cognitively unimpaired adults, and 24 patients with medical delirium. Data shown in **C**–**F** included all hip-fracture patients (SSD patients classified as not delirium in adjusted analyses). However, 16 cases did not have NfL measured, and thus, there were 434 patients with hip fracture in this analysis (not excluding SSD cases). The association between QA and NfL was stronger in patients with cognitive impairment (**E**) and high ASA scores (**F**).

Experimental studies suggest that CSF concentrations of QA above 100 nmol/L over time are associated with neurotoxicity and concentrations above 300 nmol/L are associated with acute neurotoxicity (22). The proportion of patients with such high QA concentrations was substantially larger in those with delirium, most notably in patients with medical delirium (Figure 4A). In comparison, concentrations of the endogenous NMDAR agonists glutamate and aspartate in CSF did not differ depending on delirium status in patients with hip fracture (Figure 4B). Of all the KP metabolites in serum and CSF, CSF-QA was most strongly associated with neuronal injury, as measured by NfL (Supplemental Table 5), and this finding remained following adjusted analyses (Figure 4C, with the QA association on transformed scale and Figure 4D on the original scale). The CSF-QA NfL association was strongest in patients with dementia (IQCODE ≥3.44, Figure 4E) and comorbidity (ASA III–IV, Figure 4F).

### KP metabolites and 1-year mortality following hip fracture.

**Figure 5:**
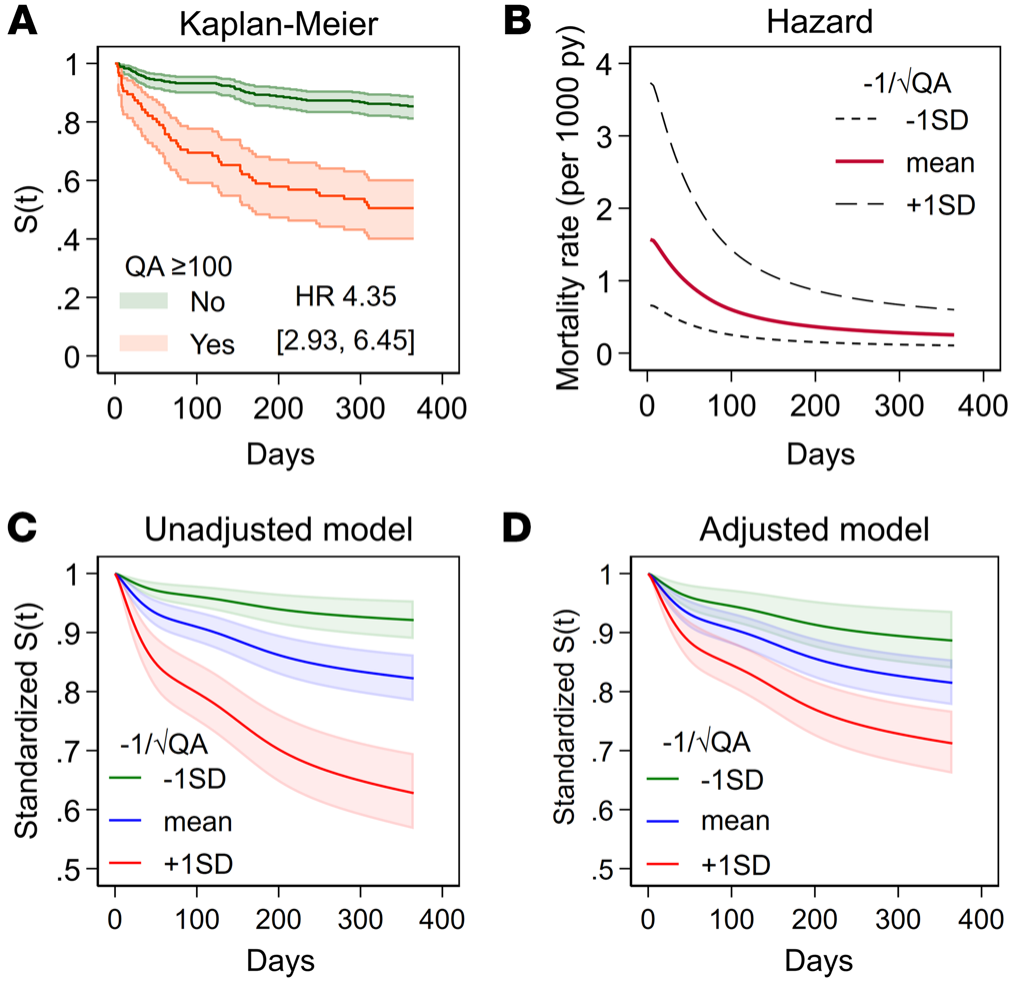
CSF-QA and 1-year survival in patients with hip fracture. Using a cutoff of 100 nmol/L, there was a clear survival benefit to patients with lower QA concentrations in univariate analyses (**A**) where the hazard for death was the highest in the initial period following the hip fracture (**B**) S(t), survival function. In unadjusted analyses, a 1 SD change in CSF-QA on the transformed scale resulted in a reduction in survival as illustrated in **C** (hazard ratio of 2.37); this was somewhat attenuated in adjusted analyses as illustrated in **D** (hazard ratio of 1.76). Of note, back-transforming the mean of QA to nmol/L results in 48.8 nmol/L, where –1 SD is 25.7 and +1 SD is 126.0. Kaplan-Meier curve in **A** and HR with 95% CI from Cox regression. **B**–**D** were estimated using a standardized parametric survival analysis where the baseline hazard was estimated using restricted cubic splines. *n* = 450; 99 events.

Among the 450 hip-fracture patients, there were 99 (22%) deaths the first year after surgery. In univariate analyses, several KP metabolites in CSF and serum were strongly associated with mortality (Supplemental Table 6). As with the association with NfL, CSF-QA was again the KP metabolite most strongly associated with mortality (Gini coefficient [GC] 0.48 indicates a strong association; Supplemental Tables 6–8). Figure 5A displays the Kaplan-Meier survival function of QA ≥ 100 nmol/L (HR of 4.35 [2.93, 6.45]; *P* < 0.001). The hazard for death was highest in the initial period following hip fracture (Figure 5B). Compared with univariate analyses (standardized survival: Figure 5C, HR: 2.37 [1.87, 3.01]), CSF-QA was attenuated in adjusted analysis (standardized survival: Figure 5D, HR: 1.76 [1.32, 2.33]) (both *P* < 0.001).

### Post hoc analyses.

Gaussian network models illustrating the relatedness of the KP metabolites by way of partial correlations revealed that serum and CSF KP metabolites were highly connected, particularly Kyn, QA, and Pic (Supplemental Figure 4). Following exploratory network analyses, no significant difference was found in network structure according to the presence of delirium (Supplemental Figure 4). However, the network structure visualizing the interrelations between the KP metabolites in CSF was significantly different from that in serum (Supplemental Figure 5). We also assessed whether the KP metabolites were more or less associated with the clinical presentation of delirium (i.e., subsyndromal, incident, or prevalent). CSF-QA was increasingly more strongly associated with delirium, moving from subsyndromal to incident and prevalent delirium (Supplemental Figure 2). Further, CSF KP metabolites, QA, and KTR were nonsignificantly more strongly associated with delirium in the absence of dementia (IQCODE < 3.44, Supplemental Figure 3).

## Discussion

Our large multicenter study of CSF in hip fracture demonstrates upregulation of the KP in the serum that was mirrored and somewhat surpassed in the CSF of patients with delirium. While malfunction of the KP is linked to both neurodegenerative diseases (such as Alzheimer’s disease [ref. 3] and Huntington’s disease [ref. 7]) and mental disorders (depression, psychosis, and schizophrenia [ref. 23]), here we provide clinical evidence linking the KP, especially higher CSF-QA, to delirium during acute illness. QA stimulates the NMDAR, implicated in the pathogenesis of psychotic disorders (24), and some delirium patients have displayed what could be potentially neurotoxic concentrations of QA. Importantly, of all the investigated KP metabolites, CSF-QA was most strongly associated with delirium, mortality, and the neuronal injury marker CSF-NfL. Our findings were substantiated by a similar pattern of changes in a group of patients with delirium triggered by a medical condition other than hip fracture. These findings suggest a possible explanation for how systemic inflammation in acute illness may cause a metabolic shift in the brain in association with delirium, neuronal injury, and mortality.

Our findings support previous reports wherein the KTR in blood has been used as an indirect measure of KP activity in the brain. In a study of 84 intensive care unit (ICU) patients, elevated plasma KP metabolites and KTR were associated with more days in delirium/coma (14). A more recent study of 130 ICU patients found significantly higher KTR in patients with delirium (*n* = 65) (15). The only other study of the KP in hip-fracture patients found higher KTR in plasma in patients with delirium before surgery (16). However, all these studies were performed using blood samples but missing CSF data; furthermore, previous studies have not performed targeted metabolomics that measures most known KP metabolites. Experimental studies investigating systemic immune activation and sepsis have demonstrated that pharmacological or genetic inhibition of IDO prevents depressive- and anxiety-like behaviors as well as cognitive deficits typically seen alongside acute inflammation (25–27). In line with this, peripheral Kyn administration induces similar deficits in a dose-dependent manner (28). Although fewer studies have focused on cognition and QA, its injection in animals’ brains has resulted in both hyperactive behavior and impairments in memory (22).

Our data suggest that concentrations of serum and CSF KP metabolites are tightly connected and that a systemic KP activation is likely to be a major contributor to brain KP metabolism (Supplemental Figure 4). This raises the question of whether adaptive mechanisms during systemic KP activation may be impaired or overwhelmed in delirium. Using ridge regression to assess highly correlated serum/CSF pairs of metabolites revealed that, although the odds of delirium were mostly due to the KTR in serum, it was almost exclusively related to CSF-QA (Supplemental Material). Further, the metabolites most strongly linked to delirium (CSF Kyn, HK, Pic, and QA) are all generated in the microglia, and microglial activation is believed to play a central role in delirium pathogenesis (9, 29). It is plausible that excessive neuroinflammation, coupled with increased systemic KP activation, contributes to the higher CSF KP metabolite concentrations observed in delirium patients.

Although there is no known threshold for QA-induced neurotoxicity in humans, we observed that QA concentrations associated with neurotoxicity in animal studies (≥100 nmol/L and ≥300 nmol/L) (22) were much more frequent in patients with delirium. QA is as potent as glutamate and aspartate in stimulating the NMDAR (22), but these amino acids were clearly not elevated in the CSF in delirium and, if anything, tended to be lower with acute illness. Also, the concentrations of KA observed in this study were much lower than reportedly required for neuroprotective antagonism of NMDAR (30). Unlike glutamate and aspartate, QA lacks a high-affinity uptake system at the synapses, making it prone to accumulation once quinolinate phosphoribosyltransferase (QPRT) becomes saturated (22). Consequently, QA is likely to act as an agonist of the NMDAR over longer time periods, a process that may eventually lead to receptor downregulation (31). This may be of significance, as NMDAR hypofunction has been linked to schizophrenia (32) and limbic encephalitis (33). QA is considered neurotoxic through several mechanisms (23), including some that are already believed to be involved in the pathogenesis of delirium, such as potentiation of the inflammatory response (34), activation of microglia (29), and excitotoxicity (9, 35). Among all the KP metabolites measured in our study, CSF-QA was most strongly associated with NfL. Further, the QA-NfL association was stronger among hip-fracture patients with cognitive impairment and high comorbidity, patients who potentially have neuronal populations that are more vulnerable to excitotoxic damage. As CSF-QA concentrations in excess of 100 nmol/L may cause cell death in the hippocampus, striatum, and cortex (22), our study proposes neurotoxic QA concentrations as a potential mechanism linking delirium and neuronal damage to subsequent cognitive decline and increased risk of dementia (36, 37).

The KP metabolites have previously been associated with all-cause mortality in population and clinical studies (38, 39). Although the mechanisms underlying these observations are not clear, they are thought to be linked to chronic inflammation, oxidative stress, and persistent immune activation. The effect sizes reported in previous studies are, however, much smaller than those we identified in our study. We believe that plausible explanations include that either QA-induced neurotoxicity contributes to mortality or that the accumulation of KP metabolites in the brain reflects a more substantial failure of homeostasis than in the blood. Regardless of the mechanism, our data suggest that KP activation in the brain following a hip fracture is an independent determinant of poor outcome. Aligning with prior experimental work, our findings suggest that the KP is a potential therapeutic target in delirium. As the most important precursor of brain KP metabolites is circulating Kyn, a natural consideration would be IDO inhibitors.

The present study has a number of strengths as well as limitations. This study benefits from the large CSF data set with a substantial number of paired CSF and serum samples. The inclusion of 2 contrast/control groups increased the validity of our findings. Delirium was assessed daily based on validated instruments administered by trained investigators. A detailed description of our diagnostic algorithm is included in Methods, as recommended for delirium studies (40). Since dementia is associated with both delirium (9) and KP metabolites (41), it was important to have information on dementia status for all patients. Due to the acute admission of patients, this was characterized using the IQCODE. Although validated and much used (42), the IQCODE is not a substitute for objective cognitive testing. Additional strengths are that all samples were analyzed simultaneously at the same laboratory by technicians blinded to all clinical data and metabolites were quantified with high precision using stable isotope–labeled internal standards (43). The KP metabolites do have inhibitory effects on the immune system, and our study is limited by a lack of data on immune function. Although repeated CSF sampling would have been desirable, such a study design would create significant ethical and practical challenges.

All in all, this study suggests that upregulation of the KP and accumulation of the potentially neurotoxic CSF-QA are strong determinants of delirium and 1-year mortality and are associated with neuronal injury. Our findings highlight a possible link among systemic inflammation, activation of the NMDAR in delirium, and neurotoxicity. Associations with mortality underscore potential pathophysiological relevance, and collectively, the results of our work should motivate studies investigating the effect of enzyme inhibitors on reducing QA formation in patients with delirium.

## Methods

### Study participants.

This was a multicenter observational study conducted at 4 hospitals in the Oslo region, Norway. Patients were included from 2009 to 2019, and CSF samples were available from 450 hip-fracture patients (224 with and 226 without delirium), 24 participants from an independent cohort of medical delirium patients, and 112 cognitively unimpaired adults scheduled for elective surgery using spinal anesthesia, for a total of 586 hospitalized participants. Of these, 338 had paired serum samples available. Delirium was assessed daily according to the DSM-5 criteria and based on a standardized procedure described previously (44). The delirium diagnosis was based on an interview with each participant supplemented by information from relatives and nurses as well as clinical notes. In short, level of arousal was assessed with the Richmond Agitation Sedation Scale (RASS) (45) and the Observational Scale of Level of Arousal (OSLA) (46), and attention was assessed using Months of the Year Backwards (MOYB), Days of the Week Backwards (DOWB), the Vigilance-A task SAVEAHAART, and counting down from 20 to 1 (47). A recall test of 3 words (different each day) was performed at each assessment. For details regarding the delirium assessments and the diagnostic algorithm, please see the Supplemental Material and Supplemental Appendix.

Patients with delirium were classified depending on delirium status at the time of CSF sampling as follows: prevalent delirium, those with delirium at the time of CSF sampling; and incident delirium, those free from delirium at the time of CSF sampling, but who developed it later. SSD was defined (in regard to patients who did not fulfill all criteria for delirium) as evidence of cognitive change, in addition to any one of the following features: (a) altered arousal, (b) attentional deficits, (c) other cognitive change, or (d) delusions or hallucinations. Preoperative ASA scores were collected from hospital records. Dementia status was assessed using IQCODE, with a score of 3.44 or more as a cutoff indicating dementia (20). In the case of missing IQCODE scores (*n* = 29 in the hip-fracture cohort), dementia status was established retrospectively using hospital records. Details regarding study participants and data collection are available in Supplemental Methods and Supplemental Table 9.

### CSF sampling and biochemical analyses.

In the hip-fracture patients and cognitively unimpaired adults, CSF was collected at the onset of spinal anesthesia before anesthetic agents were administered. The CSF of patients with medical delirium was obtained from patients who underwent diagnostic lumbar puncture due to altered mental status at a median of 1 day after CNS symptoms developed. For all participants, CSF was collected and stored in polypropylene tubes at –80°C. Prior to storage, samples were centrifuged at 2,000*g* for 10 minutes and the supernatants aliquoted. Serum was collected by venous puncture at the time of CSF sampling, centrifuged at 2,000*g* for 10 minutes, aliquoted, and stored at –80°C. Samples were sent on dry ice for biochemical analyses to the Bevital laboratory for measurement of Trp, KP metabolites, glutamate, and aspartate, and to the Clinical Neurochemistry Laboratory at Sahlgrenska University Hospital for NfL analysis. Trp, Kyn, KA, anthranilic acid (AA), HK, Pic, QA, xanthurenic acid (XA), and HAA in CSF and serum were measured using a targeted metabolomics approach by liquid chromatography–tandem mass spectrometry (LC-MS/MS) as described previously (48). The lower limit of detection (LOD) for the assay ranged from 0.01 to 8 nmol/L, and within- and between-day coefficients of variation (CVs) ranged from 3% to 8% and 4% to 10%, respectively. The KTR was calculated for both CSF and serum as 1,000 × μmol/μmol. XA and HAA concentrations in CSF were below the LOD in most samples and were not included in subsequent statistical analyses. CSF NfL concentrations were measured using a commercial ELISA (Umandiagnostics) (49). As both QA and KA engage the NMDAR, we also measured the concentrations of the other endogenous NMDAR agonists, glutamate and aspartate, in CSF and serum by gas chromatography–tandem mass spectrometry (GC-MS/MS). The LOD for the assay was 0.5 μmol/L, and within- and between-day CVs for both amino acids were 2% and 4%, respectively (Bevital laboratory).

### Statistics.

We adjusted the main analyses (i.e., the associations of the KP metabolites with delirium, NfL, and mortality in hip-fracture patients) for multiple comparisons (Benjamini-Hochberg FDR) considering *q* < 0.01 significant (adjusted *P* values, Stata package qqvalue) (50). Serum Trp followed a Gaussian distribution. All other metabolites were non-Gaussian, and we applied QQ plots according to Tukey’s ladder of powers to identify transformations that would best approximate normality (51). Metabolites (*x*) in serum, CSF KA, and AA followed log-normal distributions and were log(*x*) transformed, and the remaining CSF metabolites inverse square root transformed (*x^–1/2^*). All metabolites and continuous covariates were standardized (i.e., *z* scores).

For univariate analysis, we estimated comparable effect sizes indifferent to transformations using the AUC from the Mann-Whitney *U* test or time-dependent ROC curves using the nearest-neighbor estimation (censored data) (52). We then calculated the GC as 2 × AUC — 1, where <0.3 is approximately a small effect size, where similarly ≥0.3–0.4 is moderate and ≥0.4 is large (53), aiming to normalize the AUC so that a random classifier equals 0 and a perfect classifier equals 1 (54).

For all outcomes, we performed univariate analyses and then, subsequently, adjusted for age, sex, renal function (GFR), a binary variable for the ASA score (I–II versus III–IV), cognitive function (IQCODE cutoff 3.44), and delirium status (if not the outcome). Logistic regression was used to determine the metabolite-related odds of delirium, and similarly, Cox regression was used for the hazard rate for 1-year mortality. Flexible parametric survival analysis (Stata: stpm2) was used to estimate the hazard over time and standardized survival curves according to metabolite concentrations. Patients with SSD (*n* = 44), who could be classified neither as cases nor controls (55), were excluded from the analyses with delirium as the outcome, but investigated in subgroup analyses presented in Supplemental Material. The association between KP metabolites and NfL in hip-fracture patients was determined using Spearman’s Rho (*R*) with adjusted analyses using linear regression (log-transformed NfL). Cohorts with medical delirium and the cognitively unimpaired adults were included as reference groups. Methods used for several post hoc analyses are presented in Supplemental Methods. All analyses were conducted using R (version 4.1.2) and Stata (version 17). BioRender was used for illustrations.

### Study approval.

The study was conducted in accordance with the World Medical Association Declaration of Helsinki. The data and CSF samples were collected after informed, written consent was obtained from the patient and/or proxy (if patients were unable to consent due to cognitive impairment), as approved by the Regional Committee for Medical and Health Research Ethics in Norway (REK 2009/450, REK 2011/2052, REK 2011/2578 and REK 2016/1368).

### Availability of data.

Patient data are available with a transfer agreement.

## Author contributions

LOW initiated and designed the study, collected data from all cohorts at all sites, interpreted data, and helped revise and prepare the manuscript. CTP collected data at Akershus University Hospital, interpreted data, and helped revise the manuscript. BEN collected data, including delirium diagnostics, in hip-fracture cohorts 1 and 2, interpreted data, and helped revise the manuscript. EQP collected data for the medical delirium cohort, interpreted data, and helped revise the manuscript. NBH collected data for the cohort of cognitively unimpaired adults, interpreted data, and helped prepare the manuscript. AVI collected data for the cohort of cognitively unimpaired adults, interpreted data, and helped prepare the manuscript. BH planned CSF sampling in hip-fracture cohort 2, interpreted data, and helped revise the manuscript. KH interpreted data and helped revise the manuscript. ABK planned the study, interpreted data, and helped revise the manuscript. FF and JR collected data at Oslo University Hospital, interpreted data, and helped revise the manuscript. AG collected data at Diakonhjemmet Hospital, interpreted data, and helped revise the manuscript. PMU and AM performed biomarker analyses in serum and CSF at Bevital, interpreted data, and helped revise the manuscript. WF collected data at Bærum Hospital, interpreted data, and helped revise the manuscript. GS collected data at 1-year follow-up, interpreted data, and helped revise the manuscript. HZ analyzed NfL, interpreted data, and helped revise the manuscript. EFF provided guidance on the NAD^+^ synthetic pathway, interpreted data, and helped revise the manuscript. MM collected data at Bærum Hospital, interpreted data, and helped revise the manuscript. LMG planned the study from a biochemical standpoint, performed statistical analysis, interpreted data, and helped prepare and revise the manuscript.

## Supplementary Material

Supplemental data

ICMJE disclosure forms

## Figures and Tables

**Table 2:**
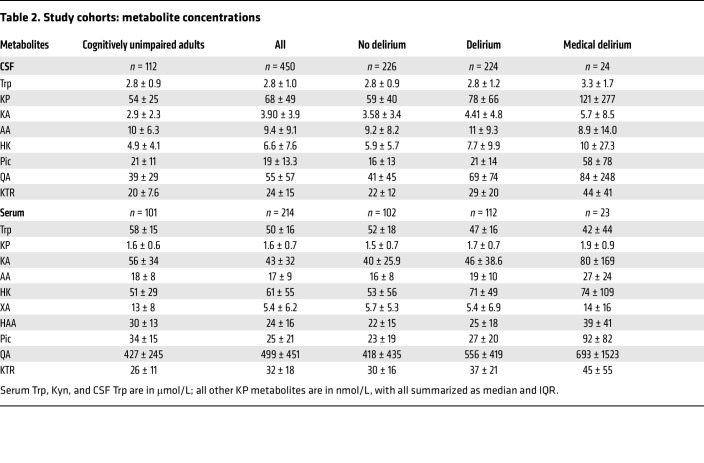
Study cohorts: metabolite concentrations

**Table 1:**
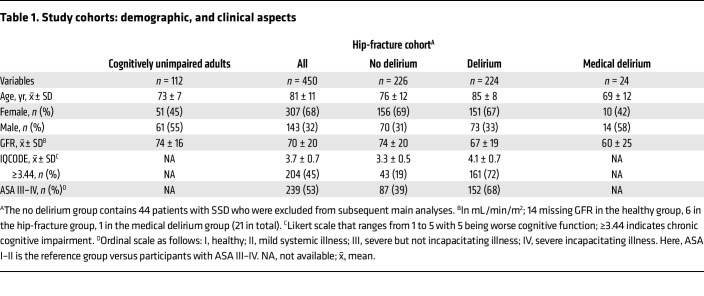
Study cohorts: demographic, and clinical aspects

## Version 1 11/21/2022

In-Press Preview

## Version 2 01/17/2023

Electronic publication
