# Supplementary material for: Cerebrospinal fluid quinolinic acid is strongly associated with delirium and mortality in hip-fracture patients
Source: J Clin Invest. 2023 Jan 17;133(2):e163472. doi: 10.1172/JCI163472 (PMC9843060; doi:10.1172/JCI163472)
Supplement: Supplemental data [file jci-133-163472-s099.pdf]

SUPPLEMENTARY MATERIAL

1.0 An overview of the kynurenine pathway

2.0 Sample characteristics and data collection

2.1 Description of the included cohorts

2.2 Renal function in the hip fracture cohort

3.0 Metabolites and delirium

3.1 Tryptophan and kynurenines in the different cohorts

3.2 Standardized effect measure: Gini Coefficient

3.3 Assessment of confounding

3.4 Multivariate logistic regression with Quinolinic acid and covariates

3.5 Cerebrospinal Fluid: Serum correlations

3.6 Ridge regression

3.7 Subgroup analyses of kynurenines and delirium

4.0 Neurofilament light chain protein (NfL)

5.0. Survival Analyses

6.0. Metabolic Networks

APPENDIX

SUPPLEMENTARY REFERENCES

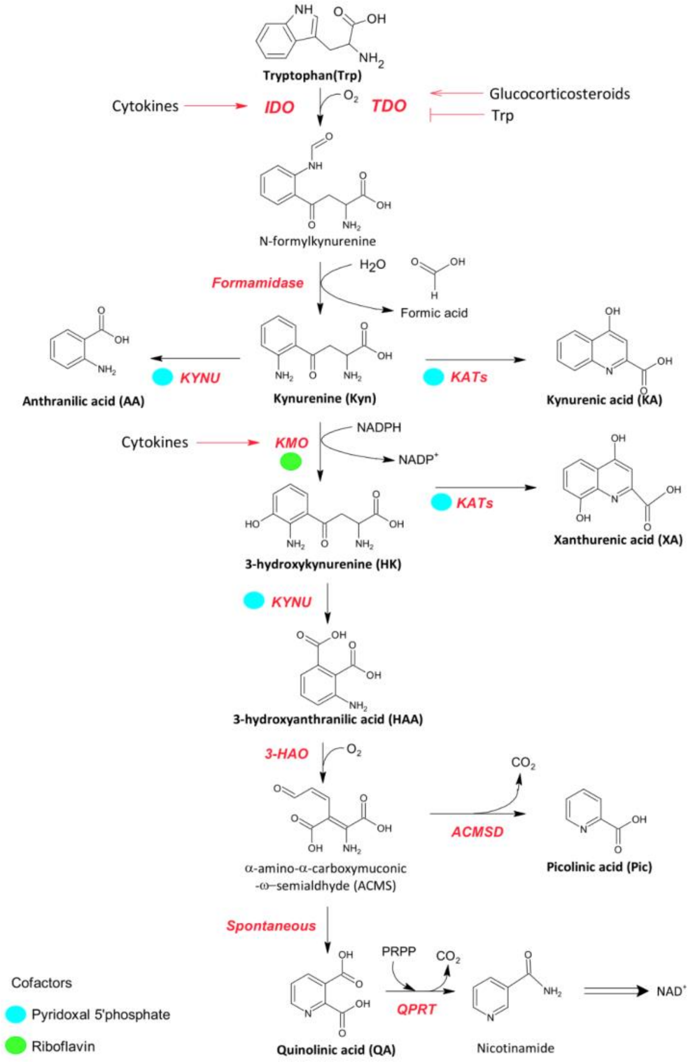

Supplementary Figure 1. An overview of the kynurenine pathway. The kynurenine pathway starts with the conversion of Trp to Kyn via N-formylkynurenine, which is regulated by the two rate-limiting enzymes TDO and IDO, expressed mainly in the liver and immune cells respectively. Cytokines, mainly interferon-γ, induce IDO. Low Trp inhibits TDO, which is activated by glucocorticosteroids. The pivotal metabolite Kyn, which crosses the blood-brain barrier, can be converted to KA or AA via KATs or KYNU, respectively. These both require pyridoxal 5'phosphate, the active form of vitamin B6, as a cofactor. However, Kyn is mainly converted to HK by the enzyme KMO, and this step too is regulated by cytokines. KMO requires the cofactors NADPH and riboflavin. HK is then converted to HAA (KYNU) and ACMS (3-HAO) which generate Pic by ACMSD or QA if ACMSD is saturated. QPRT and further steps generate NAD+ from QA (the de novo pathway of NAD+ biosynthesis). Abbreviations: 3-HAO, 3-hydroxyanthranilate 3,4 dioxygenase; ACMSD, 2-amino-3-carboxymuconate-6-semialdehyde decarboxylase; IDO, indoleamine 2,3 dioxygenase; KATs, kynurenine aminotransferase; KMO, kynurenine 3-monooxygenase; KYNU, kynureninase; NAD+, nicotinamide adenine dinucleotide; NADPH, nicotinamide adenine dinucleotide phosphate; TDO, tryptophan 2,3 dioxygenase; QPRT, quinolate phosphoribosyl transferase.

2.0 SAMPLE CHARACTERISTICS AND DATA COLLECTION

2.1. Description of the included cohorts

CSF samples were available from 450 hip fracture patients, 24 medical delirium patients, and 112 cognitively unimpaired adults, in total 586 participants. Of these, 338 had paired serum-CSF samples available. Please see figure 1 in main text for an overview.

Hip fracture patients

The hip fracture patients included in this study were sourced from two different Norwegian cohorts. The first cohort (n=104) was a single-center study with patients recruited at Oslo University Hospital between 2009 and 2012. The second cohort (n=346) was a multi-center study with patients recruited at Oslo University Hospital, Diakonhjemmet Hospital, Akershus University Hospital, and Bærum Hospital, between 2016 and 2019.

In the first cohort, delirium was assessed using the Confusion Assessment Method (CAM) (1). The study physician (LOW) or study nurse scored CAM based on a 10- to 30-minute interview with the participants, supplemented by information from relatives and nurses, as described earlier (2). Additionally, two experienced delirium researchers (LOW and BEN) independently examined hospital records in order to reveal potential episodes of delirium. In the second cohort, delirium was assessed according to the DSM-5 criteria, based on a standardized procedure described previously (3) **(see appendix for diagnostic algorithm**). Study nurses, trained in delirium assessment by the study physician (LOW), performed all assessments. The same researchers as in cohort 1 (LOW and BEN) independently assessed all available information for each patient to decide whether the DSM-5 criteria for delirium were fulfilled or not. The interrater agreement upon delirium diagnosis was excellent (kappa 0.97), with disagreements resolved through discussion. In both cohorts, delirium was assessed daily in all participants preoperatively and until the fifth postoperative day (all) or discharge (patients with delirium).

Patients with delirium were classified depending on delirium status at the time of CSF sampling: prevalent delirium – those with delirium at the time of CSF sampling, and incident delirium - those free from delirium at the time of CSF sampling, but who developed it later. Subsyndromal delirium was defined (in patients not fulfilling all criteria for delirium) as evidence of cognitive change, in addition to any one of the following features: (a) altered arousal, (b) attentional deficits, (c) other cognitive change, and (d) delusions or hallucinations.

Pre-fracture cognitive status was assessed using the Informant Questionnaire on Cognitive decline in the Elderly (IQCODE) with ≥ 3.44 as a cutoff indicating cognitive impairment (4). In the case of missing IQCODE (n=29), the pre-fracture cognitive status was established using hospital records. The American Society of Anesthesiologists Physical Status (ASA) (5)-classification level was registered from participants' medical records.

Medical delirium patients

Twenty four medical delirium patients were recruited from a prospective study conducted from 2014 and 2015 at Oslo University Hospital, aiming to identify biomarkers in patients with meningitis/encephalitis (6). The study included patients who had undergone diagnostic lumbar puncture (LP) due to suspicion of acute central nervous system (CNS) infection. None of the patients enrolled in the current study were found to have a CNS infection, and their altered mental status was considered to be delirium triggered by a medical condition (e.g. pneumonia or a urinary tract infection). Delirium was assessed either by the study physician (EQP) using CAM, or by the treating physician through clinical evaluation. Dementia status was established using the hospital records. All patients in the medical delirium group had ongoing (i.e. prevalent) delirium at the time of CSF sampling.

Cognitively Unimpaired Adults (CUA)

One hundred and twelve cognitively unimpaired persons aged > 65 years, undergoing elective gynecological, orthopedic or urological surgery in spinal anesthesia, were recruited from Oslo University Hospital and Diakonhjemmet Hospital, Oslo, between 2012 and 2013, as described

previously (7). Cognitive function was assessed through a multi-domain battery of cognitive tests prior to surgery and during annual follow up examinations. All patients were free from delirium, had a Mini-Mental State Examination (MMSE) test score ≥ 28 at baseline and did not develop dementia within the first five years of follow up.

2.2 Renal function in the hip fracture cohort

The kynurenines are mainly excreted by the kidneys and renal function is an important confounder as it affects the concentrations of circulating kynurenine metabolites (8). Creatinine, for the purpose of estimating renal function, was measured in serum by LC-MS/MS (9). Limit of detection for the assay was 0.25 µmol/L, and within- and between-day CVs ranged from 2.6% and 3.1%, respectively. Creatinine was measured in serum samples obtained at the time of CSF sampling in 239 individuals. In 186 individuals, creatinine was calculated as the average of the pre- and post-operative creatinine, whereas only pre-operative creatinine was available in 17 patients and only post-operative in 2. Estimated glomerular filtration rate (GFR) was calculated using the CDK-EPI equation (10). Six participants had missing creatinine, and GFR values were imputed as the mean.

3.0 METABOLITES AND DELIRIUM

3.1 Tryptophan and kynurenines in the different cohorts

Table 1B summarizes the metabolite concentrations in the three cohorts. Serum Trp was lower in hip fracture patients and medical delirium patients compared to the cognitively unimpaired adults, and the concentration was lowest in patients with delirium. Inversely, Kyn, HK, QA and KTR were higher in both serum and CSF in both patient groups compared to the cognitively unimpaired adults, and again the difference was largest in patients with delirium, especially in the CSF. KA was also higher in both delirium groups but only in the CSF.

The pairwise correlations between kynurenines in CSF versus serum in cognitively unimpaired adults, patients with hip fracture and medical delirium was strongest for QA and KTR and decreased in the order Pic, Kyn, HK, AA, KA and Trp (**Supplementary Table 4**).

3.2 Standardized effect measure: Gini Coefficient

The Gini Coefficient (2*area under the curve)-1 gives a standardized effect size insensitive to differences in statistical transformations. A biomarker randomly classifying groups (no effect) is equal to zero whereas a perfect classifier is equal to one, and is considered < 0.3 is a small effect size, ≥0.3 – 0.4 is moderate and ≥ 0.4 large (11). Negative values are also allowed. The results for univariate analysis comparing hip fracture patients with delirium to hip fracture patients without delirium are shown in **Supplementary Table 1**. The results show a small effect size for the association between serum Trp, Kyn, KA, AA, HK, HAA and PIC, and CSF KA, AA, HK and PIC and delirium, a moderate effect size for serum QA and KTR, and CSF Kyn and KTR. There was a large effect size for CSF QA.

Supplementary Table 1. Gini Coefficient[a] for kynurenine metabolites in patients with hip fracture and delirium (n=224) compared to patients with hip fracture without delirium (n=182).

	Serum (n = 214)	CSF (n = 406)
Trp	-0.26	0
Kyn	0.24	0.36
KA	0.18	0.22
AA	0.28	0.22
HK	0.28	0.28
XA	-0.04	
HAA	0.16	
Pic	0.16	0.26
QA	0.30	0.42
KTR	0.38	0.38

Abbreviations: AA, anthranilic acid; HAA, hydroxyanthranilic acid; HK, hydroxykynurenine; KA, kynurenic acid; Kyn, kynurenine; KTR, kynurenine:tryptophan ratio; Pic, picolinic acid; QA, quinolinic acid; XA, xanthurenic acid.
[a] Gini Coefficient (2*area under the curve – 1).
≥ 0.1 – 0.3 = small
≥ 0.3 – 0.4 = medium
≥ 0.4 = large

3.3 Assessment of confounding

We investigated the attenuation in effect sizes after adjusting for covariates by assessing the overall change-in-estimate (CIE) adjusting for all covariates and CIE per covariate to identify the variables responsible for the attenuation of the associations. Correlation coefficients or Cohen's D was also estimated between each covariate (age, gender, renal function (GFR), IQCODE and ASA score) and CSF Kyn, QA, and KTR (**Supplementary Table 2**). Age was responsible for most of the attenuation of the effect size of the kynurenine-delirium relationship. Kyn, KTR and QA in serum and CSF were positively correlated with age, inversely correlated with GFR, lower in females, and higher with morbidity (ASA score) and cognitive impairment (IQCODE). Most of these associations were somewhat stronger in serum compared to CSF.

Aging leads to multiple changes in kynurenine concentrations, including an increase in QA in both serum and CSF (12). Thus, it remains to be investigated whether aging mediates some of its inherent risk of delirium by increasing QA in the brain or whether age here acts as a typical confounder in a non-causal relationship.

Supplementary Table 2. Attenuation of effect sizes of kynurenines versus delirium by covariates

		**Serum (n = 214)**			**CSF (n = 406)**		
		R (D)[a]	p	CIE[b], %	R (D)	p	CIE[b], %
Kyn	**Overall**			-19.0			-15.8
	Age	0.30	<.001**	-20.3	0.34	<.001**	-23.2
	Female	(-0.54)	<.001**	-3.6	(-0.27)	.008*	-0.1
	GFR	-0.62	<.001**	-15.2	-0.37	<.001**	-6.3
	IQCODE[c]	(0.32)	.0.20*	-6.2	(0.29)	.004*	+6.7
	ASA III-IV	(0.44)	<.001**	-9.5	(0.52)	<.001**	-9.9
QA	**Overall**			-18.6			-20.8
	Age	0.38	<.001**	-24.4	0.40	<.001**	-26.5
	Female	(-0.37)	.007*	-2.2	(-0.29)	.005*	0.1
	GFR	-0.69	<.001**	-6.5	-0.49	<.001**	0.1
	IQCODE[c]	(0.45)	.001*	-10.8	(0.44)	<.001**	-2.8
	ASA III-IV	(0.71)	<.001**	-11.1	(0.59)	<.001**	-8.0

KTR	**Overall**			-31.7			-20.3
	Age	0.43	<.001**	-26.1	0.29	<.001**	-20.8
	Female	(-0.35)	.011*	-2.1	(-0.12)	.225	-0.2
	GFR	-0.57	<.001**	-1.0	-0.40	<.001**	-3.6
	IQCODE[c]	(0.61)	<.001**	-17.8	(0.40)	.001*	-8.4
	ASA III-IV	(0.83)	<.001**	-12.5	(0.43)	<.001**	-6.9

Abbreviations: ASA - American Society of Anesthesiologists physical status classification; GFR - glomerular filtration rate; IQCODE - Informant Questionnaire on Cognitive Decline in the Elderly; KTR - kynurenine-to-tryptophan ratio; Kyn - kynurenine; QA - quinolinic acid.

[a] Association between covariate and kynurenine. R, Pearson's correlation coefficients; (D), Cohen's D.

[b] CIE, change-in-estimate: (Odds ratio metabolite / Odds ratio metabolite + confounder) / Odds ratio metabolite + confounder

[c] IQCODE < 3.44 (reference) or ≥ 3.44 (case)

* p < 0.05, ** p < 0.001

3.4 Multivariate logistic regression with Quinolinic acid and covariates

The multivariate analysis shows that by far the strongest risk factor for delirium in patients with hip fracture was preexisting dementia (defined as IQCODE ≥ 3.44). Age and ASA score were also significantly associated with delirium, as was CSF QA (**Supplementary Table 3**).

Supplementary Table 3. CSF QA and delirium adjusted for covariates (n = 406).

	**OR**	**95% CI**	**p-value**
Age	2.10	1.48, 2.99	< 0.001**
Female	0.85	0.47, 1.54	0.432
IQCODE ≥ 3.44	11.4	6.54, 19.9	<0.001**
GFR	1.34	0.96, 1.88	0.306
ASA III-IV	2.26	1.32, 3.87	0.003*
CSF QA	1.79	1.26, 2.54	0.001*

Note: multivariate logistic regression for the listed covariates. Age, GFR and QA were standardized to a mean of 0 and a SD of 1.

Abbreviations: ASA - American Society of Anesthesiologists physical status classification; GFR - glomerular filtration rate; IQCODE - Informant Questionnaire on Cognitive Decline in the Elderly; QA - quinolinic acid.

* p < 0.05, ** p < 0.001

3.5 Cerebrospinal Fluid: Serum correlations

For Kyn, the main precursor of brain kynurenines (13), the pairwise CSF: serum Spearman correlation was 0.70 in controls, 0.53 in hip fracture patients without delirium, 0.48 in those with delirium and

0.59 in patients with medical delirium. For QA, the correlation was very strong and similar in all groups (0.80-0.82, **Supplementary Table 4**).

Supplementary Table 4. Pairwise CSF:serum Spearman correlation coefficients (R) [a]

Pairs	CUA (n=112)	Hip fracture patients			Medical delirium (n=23)
		**All (n=214)**	**No delirium (n=102)**	**Delirium (n=112)**	
Trp-Trp	0.23*	0.28**	0.17	0.35**	0.27
Kyn-Kyn	0.70**	0.55**	0.53**	0.48**	0.59*
KA-KA	0.32*	0.32**	0.35**	0.22*	0.53*
AA-AA	0.41**	0.37**	0.35*	0.39**	0.76**
HK-HK	0.41**	0.59**	0.61**	0.59**	0.67**
Pic-Pic	0.78**	0.75**	0.71**	0.75**	0.92**
QA-QA	0.82**	0.81**	0.80**	0.80**	0.80**
KTR-KTR	0.66**	0.83**	0.83**	0.78**	0.85**

Abbreviations: AA - anthranilic acid; CUA – Cognitively Unimpaired Adults; CSF - cerebrospinal fluid; KA - kynurenic acid; Kyn - kynurenine KTR - kynurenine:tryptophan ratio; HK - 3-hydroxykynurenine; Pic - picolinic acid; Trp - tryptophan; QA - quinolinic acid.
[a] 25 cases with subsyndromal delirium excluded from the analyses of hip fracture patients
* $p < 0.05$, ** $p < 0.001$

3.6 Ridge regression

For highly correlated independent variables, specialized statistical analyses are needed to adequately assess effect sizes and standard errors, one such method is ridge regression. Using ridge regression to accommodate the high collinearity expected from the strong CSF:serum correlations, we entered the CSF:serum pairs of Kyn, QA and KTR in the same model (Stata: elasticnet). Using this approach, unadjusted for covariates, the CSF vs. serum ORs were 1.56 / 1.20 for Kyn, 1.89 / 1.09 for QA and 1.30 / 1.52 for KTR. It is clear from the strong CSF:serum correlations that the systemic and CSF concentrations of kynurenines are highly dependent. However, although ridge regression is an explorative method, our findings suggest that for the potential biomarker of IDO activity, KTR,

systemic levels are important in delirium, whereas for QA, the CSF concentrations are the most important.

3.7 Subgroup analyses of kynurenines and delirium

3.7.1 Statistical methods to assess the odds for delirium stratified by subgroups

We investigated whether the associations between kynurenine metabolites and delirium was different in subgroups of patients with hip fracture who had subsyndromal delirium, incident delirium, or prevalent delirium compared to hip fracture patients without delirium. Further, we assessed whether the odds of delirium were significantly different in hip fracture patients with prevalent delirium compared to hip fracture patients with incident delirium as the reference group by introducing a statistical interaction between the kynurenine metabolite and the prevalent/incident group (the sample size of the subsyndromal delirium group was too small to assess an interaction). Using logistic regression, we adjusted for age, gender, renal function (GFR), a binary variable for ASA score (I-II versus III-IV), and cognitive function (IQCODE cutoff 3.44).

Again using logistic regression, we assessed the kynurenine-associated odds for delirium according to ASA (I-II vs. III-IV) and cognitive status (IQCODE < 3.44 vs IQCODE ≥ 3.44). These analyses were similarly adjusted for the above listed covariates, excluding the grouping variable defining strata. As these were post-hoc analyses, we focused on metabolites of interest that were most strongly associated with delirium; CSF Kyn, CSF QA, and CSF KTR.

3.7.2 Subsyndromal, incident and prevalent delirium

We aimed to assess whether selected kynurenines were associated with delirium depending on the clinical context, i.e. if symptoms were ongoing at the time of CSF sampling (prevalent delirium), developed later (incident delirium) or were mild (subsyndromal delirium). Kyn was similarly associated with prevalent delirium (OR 1.82 [1.22, 2.73], p=0.003) and incident delirium (1.60 [1.09, 2.35], p=0.017) in adjusted analyses (**Supplementary Figure 2**). However, QA was more strongly associated with prevalent delirium (OR 2.18 [1.38, 3.45], p<0.001) compared to incident delirium (OR

1.43 [0.97, 2.12], p=0.070). In contrast, KTR was more strongly associated with incident (OR 2.20 [1.62, 2.99], p=0.004) compared to prevalent (OR 1.60 [1.10, 2.33], p=0.014) delirium. All were more weakly associated with subsyndromal delirium. With one exception, the differences in the strength of association with incident compared to prevalent delirium did not reach statistical significance, although QA was significantly more strongly associated with prevalent delirium compared to incident delirium (p=0.007).

Although the sample size in the subgroups warrants caution, one may speculate that changes in CSF-KTR, a surrogate biomarker of IDO activation, may be an early event in the development of delirium, whereas QA concentrations increase later, once symptoms are established. Of note, QA increased sequentially moving from cognitively unimpaired adults, to subsyndromal delirium, incident delirium and was the highest in patients with ongoing delirium when CSF was sampled (prevalent delirium).

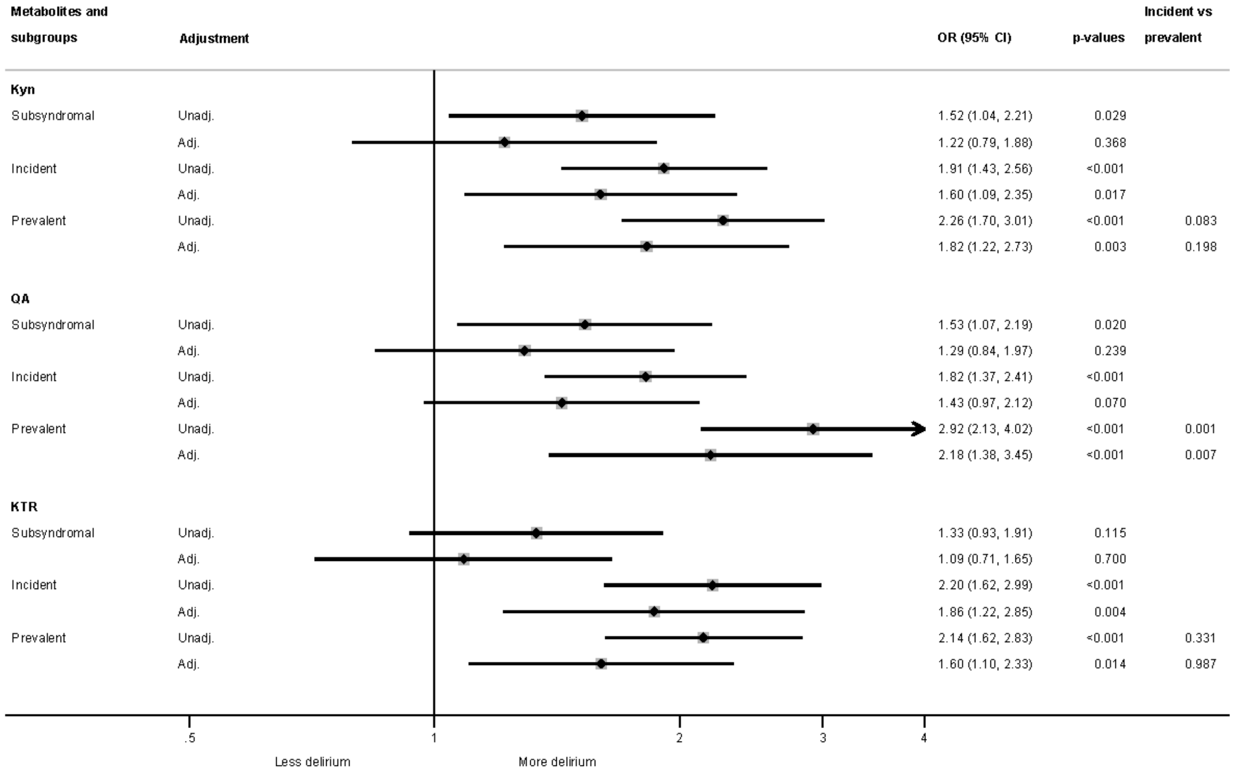

Supplementary Figure 2. Forest plot of CSF kynurenines and risk of subsyndromal, incident and prevalent delirium. Logistic regressions with either hip fracture patients with subsyndromal (n = 44), incident (n = 108) or prevalent (n = 113) delirium as the outcome and hip fracture patients without delirium (n = 182) as the reference group. Unadjusted (Unadj.) odds ratios (OR) and analyses adjusted (Adj.) for age, gender, glomerular filtration rate, ASA score (I-II vs. III-IV) and IQCODE (≥3.44 vs <3.44) as covariates. P-values indicate significance, and incident vs. prevalent (variable to the right in figure) gives a p-value for the same analysis with prevalent delirium as the outcome and incident delirium as the reference. Abbreviations: CI - 95% confidence interval; CSF - cerebrospinal fluid; KTR - kynurenine: tryptophan ratio; Kyn - kynurenine; metab - metabolites; QA - quinolinic acid.

3.7.3 Pre-operative risk score (ASA) in hip fracture patients

Adjusted for covariates, CSF-metabolite concentrations were more strongly associated with delirium in patients without severe systemic disease (ASA I-II) for Kyn (OR 1.70 [1.08, 2.67], p=0.021) and KTR (2.37 [1.44, 3.90], p<0.001) compared to patients with severe systemic disease (ASA III-IV, Kyn: 1.59 [1.04, 2.41], p=0.031, KTR: 1.46 [0.94, 2.25], p=0.089)). For QA, these were close to equivalent (ASA I-II: 1.76 [1.09, 2.82], p=0.020, ASA III-IV: 1.73 [1.09, 2.75], p=0.020). None of these differences in strength of association according to ASA status reached statistical significance (**Supplementary Figure 3**).

3.7.4 Chronic cognitive impairment

In hip fracture patients without chronic cognitive impairment, CSF Kyn (2.09 [1.30, 3.09], p=0.002), CSF QA (2.17 [1.35, 3.51], p=0.002), and CSF KTR (2.10 [1.31, 4.06], p=0.004) were all more strongly associated with delirium compared to those with chronic cognitive impairment, CSF Kyn: 1.35 [0.79, 2.29], p=0.273, CSF QA 1.38 [0.76, 2.49], p=0.290, CSF KTR 1.29 [0.77, 2.15], p=0.106). However, these differences in associations did not reach statistical significance after introducing interactions (p-value for all: >0.05, **Supplementary Figure 3**).

Overall, our results give reason to speculate whether KP metabolites may play a more important role in delirium development in patients with less comorbidity and without cognitive impairment. However, these findings were not significant and such questions will need to be addressed more robustly in future studies adequately powered for subgroup analyses.

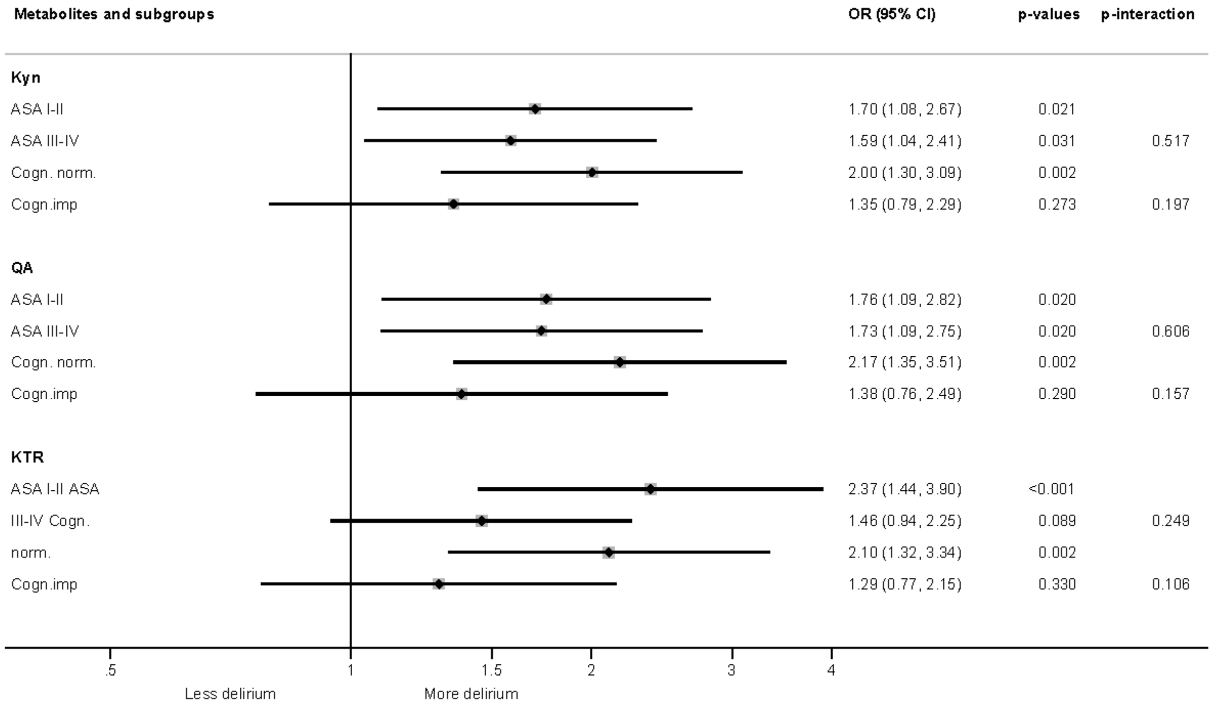

Supplementary Figure 3. Selected CSF kynurenines and risk of delirium according to comorbidity and cognitive status. Logistic regression with delirium (yes/no) as the outcome and the listed kynurenines as predictors, including the listed kynurenines stratified by groups. These were either ASA score (I-II (no delirium n = 124, delirium n = 72) vs. III-IV (no delirium n = 58, delirium n = 152) or IQCODE (cognitively normal (Cogn.norm.) < 3.44 (no delirium n = 157, delirium n = 63) vs cognitively impaired (Cogn.imp) ≥3.44 (no delirium n = 43, delirium n = 161). The analyses were adjusted for age, gender, glomerular filtration rate and ASA score (if stratified by IQCODE) or IQCODE (if stratified by ASA score). P-values indicate significance in each strata. The p-interaction indicates whether the difference in the associations between delirium and the kynurenines were significantly different according to ASA score and IQCODE (statistical interaction). Abbreviations: ASA - American Society of Anesthesiologists physical status classification; CSF - cerebrospinal fluid; IQCODE - Informant Questionnaire on Cognitive Decline in the Elderly; KTR - kynurenine:tryptophan ratio; Kyn - kynurenine; metab. - metabolites; QA - quinolinic acid.

4.0 NEUROFILAMENT LIGHT CHAIN PROTEIN

We correlated neurofilament light chain protein, a biomarker of acute neuronal injury (14), with serum and CSF kynurenines using Spearman Rho's in the whole hip fracture cohort (n = 434 (16 cased had not measured NFL), **Supplementary Table 5**). P-values were adjusted using the Stata package "qqvalue". Of all metabolites, CSF-QA was most strongly correlated with NFL (R 0.43). Further exploration of the QA-NFL association in analyses adjusted for age, gender, GFR, IQCODE ≥3.44, delirium and ASA score III-IV vs I-II are given in the main manuscript (**Figure 3**), also with analyses according to subgroups with clinical vulnerability for neuronal damage (IQCODE ≥3.44 and ASA III-IV) adjusted for the above listed covariates.

Supplementary Table 5. Spearman correlation coefficients between CSF neurofilament light chain concentrations and kynurenines in serum and CSF of all patients with hip fracture

	Serum (N = 230)		CSF (N = 436)	
	R	Q[a]	*R*	Q[a]
Trp	-0.15	0.032	0.20	<0.001**
Kyn	0.13	0.063	0.35	<0.001**
KA	0.11	0.132	0.16	0.002*
AA	0.12	0.106	0.09	0.069
HK	0.20	0.005*	0.24	<0.001**
XA	-0.04	0.626	NA	
HAA	0.03	0.653	NA	
Pic	0.10	0.137	0.17	0.001*
QA	0.21	0.004*	0.43	<0.001**
KTR	0.24	<0.001**	0.27	<0.001**

Abbreviations: AA - anthranilic acid; CSF - cerebrospinal fluid; KA - kynurenic acid; Kyn - kynurenine KTR - kynurenine:tryptophan ratio; HAA, hydroxyanthranilic acid, HK - 3-hydroxykynurenine; NA, not applicable; Pic - picolinic acid; Trp - tryptophan; QA - quinolinic acid, XA, xanthurenic acid.
[a] FDR (false discovery rate)-adjusted p-value
* <0.05, ** < 0.001

5.0 SURVIVAL ANALYSIS

Survival analysis was conducted using Cox regression in patients with hip fracture censored 365 days following hospital admission. Due to different transformations (see Statistics in the main manuscript), we could not estimate completely comparable hazard ratios as the point of the mean and the interpretation of the standard deviation varies from gaussian, log-normal and inverse square root distributions. Hence, we estimated an effect size that is robust to monotone transformations in univariate survival analysis to be able to compare the effect size of the different kynurenines. Here, we used time-dependent ROC curve estimation using the nearest neighbour algorithm and from the resulting AUC, we calculated the success rate difference ((2*AUC)-1). We applied the Stata package "stroccurve". There were 99 events in the hip fracture cohort (n = 450). Delirium, IQCODE ≥3.44 and ASA III-IV were highly predictive of mortality and as such there were only 17 events in patients without delirium (including subsyndromal delirium), 25 events in participants with IQCODE <3.44 and 27 in participants with ASA I-II. Hence, we did not perform any subgroup analysis.

In univariate analysis (**Supplementary Table 6**), the Gini coefficient indicated a weak association between survival and serum AA, XA, HAA, and PIC, and CSF KA, AA, and HK. There were moderate associations between serum HK, CSF Pic and CSF KTR with survival and strong associations with serum KA, QA, and KTR, and CSF Kyn and KA. CSF-QA predicted survival the strongest among all measured kynurenines. In Cox regression adjusted for age, gender, renal function, cognitive impairment, delirium and preoperative risk (**Supplementary Table 7**), the association between CSF QA and survival was attenuated (HR 2.37 in unadjusted analysis (**Supplementary Table 6)** and HR 1.76 in adjusted analysis (**Supplementary Table 7**)) but remained highly significant. Of note, the associations of age, GFR, ASA and especially delirium were also attenuated following the inclusion of CSF QA in the model (**Supplementary Table 7**). This suggest that confounding goes both ways where indicators of clinical severity explain some of the association between CSF QA and mortality and vice versa. The magnitude of the association between CSF QA and mortality can also be gauged using the 100 nmol/L cut-off associated with neurotoxicity or quartiles (**Supplementary Table 8**).

Supplementary Table 6. Kynurenines as predictors of one-year survival in hip fracture

	Serum (n = 239, 55 events)				CSF (n = 450, 99 events)			
	GC[a]	HR	CI	Q[b]	GC[a]	HR	CI	Q[b]
Trp	-0.14	0.80	0.62,1.03	0.091	0.08	1.11	0.94, 1.30	0.236
Kyn	0.30	1.69	1.30, 2.18	<0.001**	0.43	1.86	1.55, 2.24	<0.001**
KA	0.41	2.14	1.61, 2.83	<0.001**	0.21	1.39	1.14, 1.70	0.002*
AA	0.28	1.50	1.15, 1.96	0.004*	0.16	1.28	1.06, 1.56	0.015*
HK	0.39	1.77	1.41, 2.23	<0.001**	0.29	1.60	1.31, 1.95	<0.001**
XA	0.22	1.57	1.17, 2.12	0.004*				
HAA	0.13	1.41	1.04, 1.90	0.029*				
Pic	0.24	1.46	1.13, 1.88	0.005*	0.32	1.71	1.38, 2.13	<0.001**
QA	0.41	2.04	1.55, 2.67	<0.001**	0.48	2.37	1.87, 3.01	<0.001**
KTR	0.44	1.83	1.43, 2.34	<0.001**	0.39	1.74	1.45, 2.08	<0.001**

Analysis conducted on all hip fracture patients (n = 450) with 99 failures and censoring after 365 days following admission using time-dependent ROC curves (SRD) and cox regression (HR, CI, Q).
Abbreviations: AA - anthranilic acid; CI, 95% confidence interval; CSF - cerebrospinal fluid; KA - kynurenic acid; Kyn - kynurenine KTR - kynurenine:tryptophan ratio; HAA, hydroxyanthranilic acid, HK - 3-hydroxykynurenine; HR, hazard ratio; Pic - picolinic acid; Trp - tryptophan; QA - quinolinic acid, XA, xanthurenic acid.
[a] Gini coefficient calculated as 2*area under the curve – 1 as obtained from time-dependent ROC curve using the nearest neighbor algorithm.
[b] False discovery rate-adjusted p-value
* q-value < 0.05, ** q-value < 0.001

Supplementary Table 7. Multivariate survival analysis[a] with covariates and quinolinic acid

	**Covariates only**			**Covariates and QA**		
	HR	CI	p	HR	CI	p
Age	1.40	1.02, 1.92	0.038*	1.27	0.92, 1.74	0.143
Female	0.54	0.36, 0.81	0.003*	0.59	0.39, 0.87	0.011*
GFR	0.78	0.62, 0.97	0.027*	0.93	0.73, 1.17	0.524
IQCODE ≥3.44	2.14	1.30, 3.51	0.003*	2.17	1.32, 3.55	0.002*
Delirium	2.91	1.63, 5.20	<0.001**	2.60	1.46, 4.63	0.001*
ASA III-IV	1.53	0.98, 2.41	0.064	1.36	0.86, 2.16	0.184
QA				1.76	1.32, 2.33	<0.001**

Abbreviations: ASA, American Society of Anesthesiology Physical Status Classification System; GFR, glomerular filtration rate; IQCODE, Informant Questionnaire on Cognitive Decline in the Elderly; QA, quinolinic acid.
[a] Multivariate cox regression with all hip fracture patients (n = 450) with 99 failures prior to censoring after 365 days following admission.
*p-value < 0.05, **p-value < 0.001

Supplementary Table 8. CSF quinolinic acid and mortality using cut-off at 100 nmol/L and quartiles in hip fracture patients (n=450).

	Unadjusted analyses			Adjusted analyses[a]		
	HR	CI	p	HR	CI	p
QA100	4.35	2.93, 6.46	<0.001**	2.65	1.71, 4.10	<0.001**
QA quartiles						
2 vs 1	1.60	0.69, 3.71	0.269	1.12	0.47, 2.66	0.793
3 vs 1	3.05	1.42, 6.54	0.004*	1.56	0.70, 3.49	0.279
4 vs 1	7.43	3.66, 15.1	<0.001**	3.18	1.44, 7.04	0.004*

Abbreviations: CI, 95% confidence interval;; HR, hazard ratio; QA - quinolinic acid.
[a] See Supplementary Table 7 for statistical method and covariates.
* p-value < 0.05, ** p-value < 0.001

6.0 METABOLITE NETWORKS

Using the R-package Q-graph (15), we generated a Gaussian graph which displays partial correlations (based on a Pearson's correlation matrix) which is a recommended method for assessing metabolic networks (16). Retained on the graph are those who remained significant following FDR-adjustment. A Gaussian graph was generated according to group status for cognitively unimpaired adults, hip fracture patients without delirium and hip fracture patients with delirium (**Supplementary Figure 4**). These were based on patients with both serum and CSF samples available. Notably, serum Trp, HK, and Kyn are placed centrally in the network and are known to cross the BBB (13). From inspection of the proximity of nodes, it is clear that serum and CSF concentrations of QA and Pic, are closely related in all cohorts, as was Kyn. This suggests that both Pic and QA could be measured in the periphery and give a relatively good indication of concentrations in the CNS. This is important since CSF sampling often is not possible in delirium studies. The close proximity of serum and CSF QA in the network, warrants suspicion as to whether QA, like Trp, Kyn and HK, also crosses the BBB or whether their metabolic activity is closely co-regulated outside and inside the CNS. In rats, QA crosses the BBB minimally by passive diffusion (17). CSF and serum kynurenine metabolism gives rise to a dense and likely biologically highly interrelated network. Since delirium obviously is affecting the brain, and is triggered by systemic events, the close relationship between systemic and CSF concentrations of kynurenine metabolites would be a prerequisite if the kynurenines should have relevance in the pathogenesis of delirium. We used the R-package "NetworkComparisonTest" to compare the partial correlation networks between cognitively unimpaired adults and hip fracture with and without delirium. Edge-comparisons were FDR-adjustment and we used 500 permutations for comparing differences. We did not detect significant differences between the groups (illustrated in **Supplementary Figure 4**) in network structure invariance, global strength invariance, and differences in edges (data not shown). This suggests that the properties of these highly interrelated systemic and brain metabolic networks are not altered or adapted to very high concentrations of neuroactive kynurenines in the brain of patients with delirium.

Using the same NetworkComparisonTest, we explored the comparison of serum versus CSF Gaussian graphs (**Supplementary Figure 5**) using metabolites from all cognitively unimpaired adults and hip fracture patients to increase statistical power. The serum and CSF networks were overall significantly different (Network invariance test $p < 0.001$, global strength invariance test $p = 0.024$ with 15 out of 21 edges significantly different). Most notably, Trp was less negatively correlated with the end-product QA in the CSF, HK was more strongly correlated with QA in the CSF whereas KA was more closely correlated with Kyn in the CSF, perhaps somewhat reflecting the segregated metabolism of kynurenines in the brain where HK gives rise to QA in microglia whereas KA is generated in astrocytes (13). This shows that although the serum and CSF metabolites are often strongly correlated, there are several differences between these metabolic network signatures in serum and CSF, highlighting the importance of investigating the CSF in studies aiming to understand the pathophysiology of delirium.

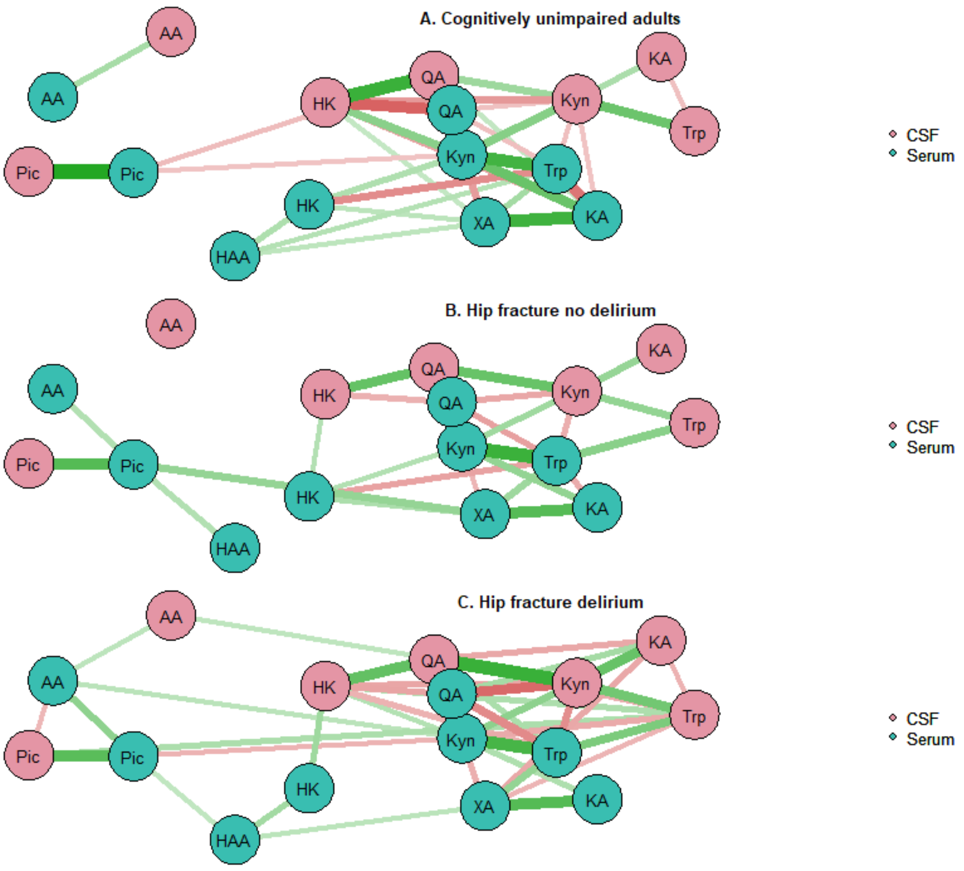

Supplementary Figure 4. Gaussian graph of serum and CSF kynurenine metabolites by groups. Green lines indicate positive partial correlations (edges) and red indicates negative. The position of each kynurenine is based on its interconnectedness (i.e. partial correlations) with all other metabolites. The overall layout has been averaged for all groups so that group comparison should be not be based on node position and layout but on the thickness of the edges. We did not detect significant group-differences in networks using the NetworkComparisonTest (see 6.0 Metabolite Networks). Abbreviations: AA, anthranilic acid; CSF, cerebrospinal fluid; HAA, 3-hydroxyanthranilic acid; HK, 3-hydroxykynurenine; KA, kynurenic acid; KYN - kynurenine; Pic, picolinic acid; QA, quinolinic acid; Trp, tryptophan.

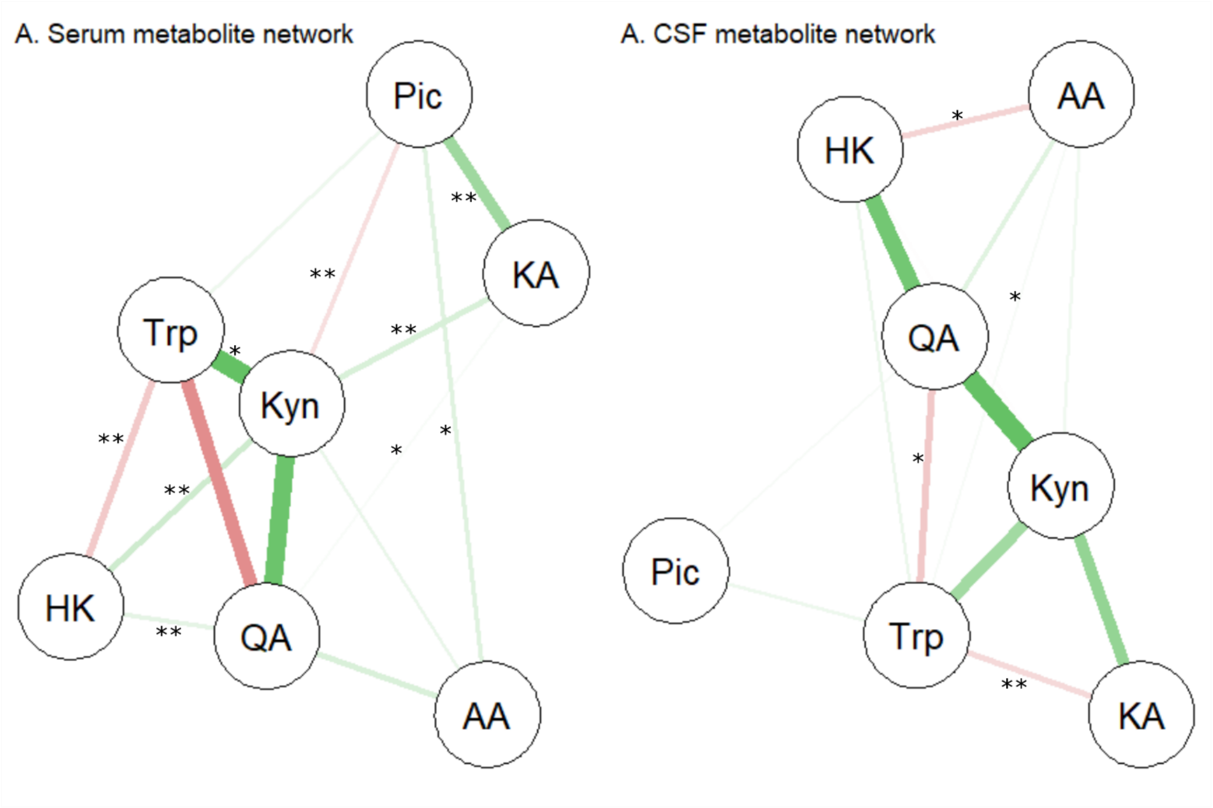

Supplementary Figure 5. Gaussian graph of serum vs. CSF using data from cognitively unimpaired adults and all patients with hip fracture combined (serum n=315, CSF=562). Green lines indicate positive partial correlation, red lines indicate negative partial correlation. * or ** indicates differences in edges between the groups (i.e. partial correlations) with an FDR-adjusted p-value < 0.05 or 0.001. Differences between the groups was tested using the NetworkComparisonTest (see text 6.0 Metabolite Networks) and are indicated on the serum graph, or if the edge was not visible there, on the CSF graph. Node position and layout was allowed to differ between the groups of metabolites. Their meaning is identical. Of the most significant differences, Kyn was more strongly related to KA in the CSF, less related to HK and Pic (negative in serum). QA was not differentially related to Kyn in the CSF, but more positively associated with HK, where HK and QA were less negatively associated with Trp in the CSF whereas Trp was negatively associated with KA in the CSF but not in serum. Abbreviations: AA, anthranilic acid; CSF, cerebrospinal fluid; HAA, 3-hydroxyanthranilic acid; HK, 3-hydroxykynurenine; KA, kynurenic acid; Kyn, kynurenine; Pic, picolinic acid; QA, quinolinic acid; Trp, tryptophan.

APPENDIX

<table>
<tr><td colspan="4">Supplementary Table 9. Diagnostic algorithm for DSM-5 delirium</td></tr>
<tr><td rowspan="2">DSM-5 criteria</td><td rowspan="2">Tests to be performed or information needed</td><td colspan="2">DSM-5 criteria fulfilled?</td></tr>
<tr><td>YES</td><td>NO</td></tr>
<tr><td>A. Disturbance in attention (i.e., reduced ability to direct, focus, sustain, and shift attention) and awareness (reduced orientation to the environment).</td><td>
<table>
<tr><th>Evaluation</th><th>TEST</th><th>Cut off (definition of inattention)</th></tr>
<tr><td>Daily</td><td>SAVEAHAART</td><td>2 or more errors</td></tr>
<tr><td>Daily</td><td>Days of the week in reverse order</td><td>any error</td></tr>
<tr><td>Daily</td><td>Months of the year in reverse order</td><td>unable to reach July</td></tr>
<tr><td>Daily</td><td>Count backwards from 20 to 1</td><td>any error</td></tr>
</table>
<u>Observation (by the examiner):</u>
Distractibility. Comprehension. Tendency to lose the thread of conversation. RASS. OSLA.</td><td></td><td></td></tr>
</table>

B. The disturbance develops over a **short period of time** (usually hours to<br>a few days), represents a **change** from baseline attention and awareness, and<br>tends to **fluctuate** in severity during the course of a day.	Informant history from patient´s carers and nursing staff or case notes.<br><br><u>Questions to carer/ nursing staff or derived from clinical notes:</u><br>Has there been a sudden change in the patient´s mental state?<br>Does the patient seem to be better at any period in the day compared to other times?<br>Has the level of consciousness been altered (drowsy/ not interacting or agitated)?<br>Sleep-wake cycle disturbances?		
C. An additional disturbance in **cognition** (e.g., memory deficit, disorientation, language, visuospatial ability, or perception).	<u>Questions to the patient:</u><br>Orientation to time, place and person<br>Recall (3 words, different list of words each day)<br>Why are you in hospital? Will a stone float in water? Are there fish in the sea? (any error = disorganised thinking)<br>Patients asked daily if they had experienced episodes of hallucinations/illusions.<br><br><u>Questions to carer/ nursing staff or derived from clinical notes:</u><br>Have there been any…:		

	Perceptual disturbances? Sleep-wake cycle disturbances? Memory disturbances? Psychotic symptoms? Psychomotor abnormalities?		
D. The disturbances in criteria A and C are **not explained by another preexisting, established, or evolving neurocognitive disorder** and do not occur in the context of a severely reduced level of arousal, such as coma.	Information from history/chart/clinical assessment		
E. There is evidence from the history, physical examination, or laboratory findings that the disturbance is a direct **physiologic consequence** of another medical condition, substance intoxication	Information from history/chart/ clinical assessment		

or withdrawal (i.e., because of a drug of abuse or to a medication), or exposure to a toxin or is because of multiple etiologies.			
**Delirium** based on the tests and information above?	All DSM-5 criteria fulfilled	Yes □	No □
**Subsyndromal delirium** based on the tests and information above?	DSM-5 criteria not fulfilled, but evidence of change, in addition to any one of these: (a) altered arousal, (b) attentional deficits, (c) other cognitive change, (d) delusions or hallucinations	Yes □	No □

SUPPLEMENTARY REFERENCES

1. Inouye SK, van Dyck CH, Alessi CA, Balkin S, Siegal AP, and Horwitz RI. Clarifying confusion: the confusion assessment method. A new method for detection of delirium. *AnnInternMed.* 1990;113(12):941-8.

2. Watne LO, Torbergsen AC, Conroy S, Engedal K, Frihagen F, Hjorthaug GA, et al. The effect of a pre- and postoperative orthogeriatric service on cognitive function in patients with hip fracture: randomized controlled trial (Oslo Orthogeriatric Trial). *BMC Med.* 2014;12(1):63.

3. Pollmann CT, Mellingsæter MR, Neerland BE, Straume-Næsheim T, Årøen A, and Watne LO. Orthogeriatric co-management reduces incidence of delirium in hip fracture patients. *Osteoporos Int.* 2021.

4. Jorm AF. The Informant Questionnaire on cognitive decline in the elderly (IQCODE): a review. *International psychogeriatrics.* 2004;16(3):275-93.

5. Mayhew D, Mendonca V, and Murthy BVS. A review of ASA physical status - historical perspectives and modern developments. *Anaesthesia.* 2019;74(3):373-9.

6. Quist-Paulsen E, Aukrust P, Kran AB, Dunlop O, Ormaasen V, Stiksrud B, et al. High neopterin and IP-10 levels in cerebrospinal fluid are associated with neurotoxic tryptophan metabolites in acute central nervous system infections. *J Neuroinflammation.* 2018;15(1):327.

7. Idland AV, Sala-Llonch R, Watne LO, Brækhus A, Hansson O, Blennow K, et al. Biomarker profiling beyond amyloid and tau: cerebrospinal fluid markers, hippocampal atrophy, and memory change in cognitively unimpaired older adults. *Neurobiol Aging.* 2020;93:1-15.

8. Theofylaktopoulou D, Midttun Ø, Ulvik A, Ueland PM, Tell GS, Vollset SE, et al. A community-based study on determinants of circulating markers of cellular immune activation and kynurenines: the Hordaland Health Study. *Clin Exp Immunol.* 2013;173(1):121-30.

9. Midttun Ø, Kvalheim G, and Ueland PM. High-throughput, low-volume, multianalyte quantification of plasma metabolites related to one-carbon metabolism using HPLC-MS/MS. *Anal Bioanal Chem.* 2013;405(6):2009-17.

10. Levey AS, Stevens LA, Schmid CH, Zhang YL, Castro AF, 3rd, Feldman HI, et al. A new equation to estimate glomerular filtration rate. *Annals of internal medicine.* 2009;150(9):604-12.

11. Cattaneo M, Malighetti P, and Spinelli D. Estimating Receiver Operative Characteristic Curves for Time-dependent Outcomes: The Stroccurve Package. *The Stata Journal.* 2017;17(4):1015-23.

12. de Bie J, Guest J, Guillemin GJ, and Grant R. Central kynurenine pathway shift with age in women. *Journal of neurochemistry.* 2016;136(5):995-1003.

13. Schwarcz R, Bruno JP, Muchowski PJ, and Wu HQ. Kynurenines in the mammalian brain: when physiology meets pathology. *Nat Rev Neurosci.* 2012;13(7):465-77.

14. Khalil M, Teunissen CE, Otto M, Piehl F, Sormani MP, Gattringer T, et al. Neurofilaments as biomarkers in neurological disorders. *Nature reviews Neurology.* 2018;14(10):577-89.

15. Epskamp S, Cramer AO, Waldorp LJ, Schmittmann VD, and Borsboom D. qgraph: Network visualizations of relationships in psychometric data. *Journal of statistical software.* 2012;48:1-18.

16. Krumsiek J, Suhre K, Illig T, Adamski J, and Theis FJ. Gaussian graphical modeling reconstructs pathway reactions from high-throughput metabolomics data. *BMC Syst Biol.* 2011;5(1):21.

17. Fukui S, Schwarcz R, Rapoport SI, Takada Y, and Smith QR. Blood-brain barrier transport of kynurenines: implications for brain synthesis and metabolism. *J Neurochem.* 1991;56(6):2007-17.
